# Effectiveness of and Mechanisms of Change in a Self-Help Web- and App-Based Resilience Intervention on Perceived Stress in the General Working Population: Randomized Controlled Trial

**DOI:** 10.2196/78335

**Published:** 2026-01-05

**Authors:** Sandy Hannibal, Dörte Behrendt, Michèle Wessa, Sarah K Schäfer, Nina Dalkner, Dirk Lehr

**Affiliations:** 1 Department of Health Psychology and Applied Biological Psychology, Institute of Sustainability Psychology, Leuphana University of Lüneburg, Universitätsallee 1, Lüneburg, 21335, Germany, 49 41316772378; 2 Department of Neuropsychology and Psychological Resilience Research, Central Institute of Mental Health, Mannheim, Germany; 3 Research Division Cancer Survivorship and Psychological Resilience, German Cancer Research Center (DKFZ)-Hector Cancer Institute at the University Medical Center Mannheim, Mannheim, Germany; 4 Division Cancer Survivorship and Psychological Resilience, German Cancer Research Center (DKFZ) Heidelberg, Heidelberg, Germany; 5 Department of Clinical Psychology and Psychotherapy for Children and Adolescents, Technische Universität Braunschweig, Braunschweig, Germany; 6 Leibniz Institute for Resilience Research, Mainz, Germany; 7 Division of Psychiatry and Psychotherapeutic Medicine, Medical University of Graz, Graz, Austria

**Keywords:** stress, resilience factor, resilience mechanism, resilience training, digital mental health intervention, internet-based intervention, mobile intervention, occupational eMental health, prevention, RCT, randomized controlled trial, mobile phone

## Abstract

**Background:**

Promoting individual resilience—that is, maintaining or regaining mental health despite stressful circumstances—is regarded as an important endeavor to prevent mental illness. However, digital resilience interventions designed to enhance mental health have yielded mixed results. Such heterogeneous effects reflect a variety of unsolved conceptual challenges in interventional resilience research. These range from grounding interventions in resilience frameworks, using theory or targeting etiologically important resilience factors as intervention content, to a lack of knowledge about the mechanisms underlying effects, and using techniques specifically developed to foster psychosocial resources. The web- and app-based resilience intervention RESIST was designed to address these challenges, mainly by using both the Positive Appraisal Style Theory of Resilience as its theoretical foundation and interventional techniques from Strengths-Based Cognitive Behavioral Therapy.

**Objective:**

This study’s primary aim was to evaluate the effectiveness of RESIST on perceived stress in a general working population as a means of universal prevention, relative to a waitlist control group. A secondary study aim was to explore the resilience factors of self-efficacy, optimism, self-compassion, and perceived social support, the intervention targets as potential mediators of its effect on stress and self-perceived resilience.

**Methods:**

In total, 352 employees were randomly assigned to either a self-help version of RESIST or a waitlist control group. Data were collected via the web at baseline, postintervention, and at 3- and 6-month (intervention group [IG] only) follow-ups. The primary outcome was perceived stress, measured with the Perceived Stress Scale-10. Secondary outcomes included self-perceived resilience, the resilience factors targeted, and other mental and work-related health outcomes.

**Results:**

The IG reported significantly less stress than controls postintervention (*Δ*=−3.14; *d*=−0.54, 95% CI −0.75 to −0.34, and *P*<.001) and at 3-month follow-up (*Δ*=−2.79; *d*=−0.47, 95% CI −0.71 to −0.22, and *P*=.002). These improvements in the IG were maintained at 6-month follow-up. Favorable between-group differences also were detected for self-perceived resilience and the resilience factors. IG participants completed on average 2.2 (SD 2.3) web-based sessions and used the app’s core feature a median of 14 times (IQR 4.00-33.75, range 1-220). The positive effects of the intervention on stress and resilience were primarily mediated by changes in optimism and self-compassion. No evidence was found that self-efficacy and social support also acted as mediators.

**Conclusions:**

In a sample of employees experiencing heightened work-burden levels, RESIST was effective in reducing perceived stress and increasing self-perceived resilience as well as the targeted resilience factors. Mediation analyses suggested that developing a positive future outlook and a self-compassionate attitude toward oneself may be key drivers to enhance resilience. Changing the quality of social relationships and strengthening the belief in one’s abilities may require more time, the involvement of others, or personal support from an eCoach to ensure sufficient learning opportunities.

## Introduction

### Background

Stressors are an integral part of life. By challenging an individual to adapt to new circumstances, they provide opportunities for growth and development. Most people can maintain or quickly regain good mental health despite experiencing stress—a phenomenon referred to as resilience [1-3]. However, when adaptation fails, stressors can contribute to the development of stress-related mental disorders [4]. Given that disorders such as depression and anxiety rank among the top 25 leading causes of global health-related burden [5,6], finding effective strategies to help individuals adapt to stress and prevent mental illness is of high importance. The promotion of individual resilience can, therefore, be an important endeavor to mitigate the potentially harmful effects of stress for individuals and, in turn, societies.

Resilience, however, is a complex phenomenon and the subject of extensive research. Resilience research has not yet reached consensus on how to define or conceptualize it. Although definitions vary, recent conceptual approaches, such as the Positive Appraisal Style Theory of Resilience (PASTOR) [2,3], converge on the idea that resilience reflects a dynamic, active, and multidimensional process of adaptation to stressors. Since this process is potentially modifiable, this opens avenues for resilience-promoting interventions.

### Evidence for Resilience-Promoting Interventions

Published evidence on the effectiveness of both nondigital and digital resilience interventions, however, remains mixed, with highly heterogeneous effects observed between primary studies and inconsistent conclusions drawn in systematic reviews and meta-analyses [7-11]. Concerning digital interventions, one meta-analysis identified small-to-moderate effects on self-perceived resilience, but not on stress reduction [7], while another found no effect on self-perceived resilience [8]. Conversely, a recent meta-analysis by Schäfer et al [11] detected small, favorable effects both on perceived stress and self-perceived resilience. Favorable effects were also found for various resilience factors, including self-compassion, optimism, and social support [11]. Overall heterogeneity in the literature may stem from the general lack of consensus on what constitutes a resilience intervention [9,12], resulting in inconsistent inclusion and exclusion decisions between reviews.

### Classification of Resilience Interventions

#### Overview

One approach has been to classify any intervention as a resilience intervention, regardless of more specific characteristics, as long as its primary objective is to modify resilience-related outcomes. These outcomes can include both self-perceived resilience, operationalized as the perceived ability to bounce back and recover from stress, or via related mental health outcomes (eg, stress and well-being).

An alternative approach is to define resilience interventions based on more specific intervention characteristics, including (1) the theory or rationale underlying the intervention; (2) the intervention’s content; (3) the interventional techniques applied; and (4) the timing of the intervention relative to stressor exposure. These dimensions contribute to significant variability in the design of resilience interventions and represent a variety of unsolved conceptual challenges in interventional resilience research.

#### Underlying Theory

Concerning underlying theories and rationales behind interventions, many resilience interventions take a pragmatic, atheoretical approach, often without reference to any specific theoretical foundation. Conversely, some interventions are informed by established theories drawn from related domains, which are then applied to the context of resilience [12]; for example, the Transactional Model of Stress [13]. Notably rare, however, are interventions explicitly grounded in a specific theory of resilience or a genuine resilience framework. As introduced earlier, PASTOR [2,3] provides such a framework. It emphasizes that resilience as an outcome from a dynamic, active, and multicausal process of stressor adaptation is shaped by both internal and external resilience factors. The theory further highlights that these resilience factors might exert their protective effects through common cognitive appraisal processes, and that the core mechanism underlying resilience may be a positive appraisal style of stressors, that is, a general tendency to appraise stressors in a nonnegative, nonthreatening, but positive way.

#### Intervention Content

Further, an intervention may be considered a resilience intervention based on its content, regardless of the outcomes studied. The most common approach involves targeting intervention content toward building or strengthening certain resilience factors. These factors predominantly consist of a broad range of psychosocial resources, including self-efficacy, locus of control, problem-solving, optimism, cognitive flexibility, mindfulness, self-compassion, and perceived social support [7-9]. Such resources have been shown to be statistically associated with resiliency outcomes and are thought to facilitate adaptive responses to stress [2], thereby helping individuals to bounce back from adversity. Consistent with the idea that access to a broad repertoire of resources facilitates flexible responses to diverse challenging situations [14], resilience interventions should, nonetheless, aim to target multiple resilience factors rather than a single one. For instance, enhancing self-efficacy may have limited value in situations where an individual is unable to influence outcomes through their own actions. However, certain resilience factors are also commonly targeted in other well-established intervention approaches, including problem-solving approaches [15] and mindfulness-based practices [16]. In contrast, if resilience interventions are categorized as a distinct class of intervention, it is essential to delineate the specific factors that should be targeted to meaningfully differentiate them from other existing approaches. In this regard, the resilience factors addressed within such interventions may be those that reflect more specific and overarching resilience processes and include, for example, factors that may serve as correlates or indicators of higher-order resilience mechanisms, such as a positive appraisal style [2].

The web- and app-based resilience training RESIST is an example of an intervention that is grounded in PASTOR [2], as the intervention’s underlying theoretical framework. It aims to foster a set of specific resilience factors, namely self-efficacy, optimism, self-compassion, and social support, associated with a positive appraisal style as a higher-order resilience mechanism [17]. A pilot randomized controlled trial (RCT) has already demonstrated its effectiveness [17], providing preliminary evidence that an intervention based on these principles might be a promising approach to fostering self-perceived resilience.

#### Mechanisms of Change

Despite certain resilience factors being targeted within given interventions, it nonetheless remains largely unknown whether favorable intervention outcomes can be directly attributed to increases in these resilience factors. In such cases, the targeted resilience factors would function as mediators of the intervention, that is, the intervening variables that statistically account for the relationship between an intervention and its outcome [18]. They may therefore point to mechanisms of change through which the intervention achieves its effects [19]. Notwithstanding the growing number of resilience interventions, empirically based understanding about their mechanisms of change remains scarce [11]; with only a few published studies, often inadequately powered, having explored this [20,21]. Examining the mechanisms underlying resilience interventions could offer valuable insights into core processes promoting resilience. Such insights could help to identify the most appropriate selection of resilience factors for interventions to target, ultimately contributing to their optimization and increased effectiveness.

#### Interventional Techniques

Concerning the intervention techniques used, which determine how intervention content is delivered, there appears to be a disconnect between the psychosocial resources targeted by resilience interventions and the use of techniques explicitly designed to foster such resources (eg, keeping a diary with a focus on successful experiences to foster resilience by enhancing self-efficacy). Indeed, many resilience interventions adopt a problem-reducing approach [22], rooted in psychotherapeutic approaches via, for example, cognitive restructuring techniques. One contrasting example of an approach explicitly developed to foster individuals’ protective resources to build resilience is Strengths-Based Cognitive Behavioral Therapy (Strengths-Based CBT) by Padesky and Mooney [23]. Among the first of its kind, the RESIST intervention applied this approach as the intervention technique used it to target the resilience factors as intervention content [17]. Since RESIST targets employees experiencing workplace stressors, it can be classified, in terms of the timing of the intervention relative to stressor exposure (4), as an intervention for use during stressor exposure [12].

### Aims of This Study

Building on the findings of the pilot study assessing RESIST in an indicated preventive setting [17], we conducted a sufficiently powered study in the general working population to compare the effectiveness of RESIST against a waitlist control group (WL) in reducing subjective stress levels, which served as the primary outcome. We conducted further exploratory analyses to examine its effects on various secondary outcomes, including self-perceived resilience and the resilience factors targeted within the intervention. In light of the urgent need to address the gap in knowledge regarding resilience interventions’ mechanisms of change, we also examined, as a secondary aim, the mediating effects of the resilience factors emphasized in RESIST on stress and self-perceived resilience.

## Methods

### Study Design

This study was conducted as a two-arm RCT from November 2019 to August 2021 in Germany. To investigate the intervention effects on the primary outcome of perceived stress, participants were randomly assigned either to the intervention group (IG) with immediate access to the resilience intervention RESIST in the form of a self-help format or to a WL whose members were offered the resilience intervention after 3 months of waiting time.

The study is reported in compliance with the CONSORT-eHEALTH (Consolidated Standards of Reporting Trials of Electronic and Mobile Health Applications and Online Telehealth) guidelines for improving and standardizing the reporting of web-based and mobile health interventions [24] and following the recommendations of Chmitorz et al [12] for reporting trials on resilience interventions. The intervention itself is reported in accordance with the TIDieR (Template for Intervention Description and Replication) checklist [25]; an overview of the checklist, along with information on where to find the corresponding details in the main text, is provided in Multimedia Appendix 1.

### Ethical Considerations

This study received ethical approval from the Ethics Committee of Leuphana University of Lüneburg. Before initiating subject enrollment, this study was registered at the German Clinical Trials Registry (DRKS00017605), a primary registry of the World Health Organization. All participants provided written informed consent before taking part in this study. Participants were informed that they could withdraw their consent at any time and request the deletion of their data without experiencing any negative consequences.

All data were deidentified before analysis; personal identifiers were replaced with unique codes, and the coding key was stored securely and separately. Access to identifiable data was restricted to authorized members of the research team. Participation in this study was entirely voluntary and not financially compensated.

### Participant Recruitment

This study was conducted in a universal preventive setting, addressing the general working population. Participants were recruited via (1) articles on resilience containing a link to the study’s landing page in two well-known popular German science magazines (“Spektrum der Wissenschaft” and “Spektrum der Psychologie”); (2) newsletters from several statutory German health insurance companies (eg, Daimler BKK) that included a section referring to this study; and (3) social media posts on various platforms (Xing [New Work SE], Instagram [Instagram from Meta], and Facebook [Meta]).

Individuals who entered their email address in a form on this study’s landing page or contacted us directly via email received detailed information about this study and were provided access to an online screening questionnaire via email. Inclusion criteria for study participation were (1) being aged at least 18 years, (2) having employment (part-time or full-time), (3) having steady access to the internet, and (4) possessing a smartphone. Exclusion criteria were (1) having an earlier or current diagnosis of a severe psychological disorder (eg, psychosis); (2) undergoing current psychotherapy, including being on a waiting list for or having the intention to receive psychotherapy; (3) current participation in another resilience intervention or stress management program; (4) changes in current medication for a stress disorder, anxiety disorder, or depression within the past four weeks; and (5) current suicidal ideation (agreeing with either the statement “I would like to kill myself” or “I would kill myself if I had the chance” on the related item in the Beck Depression Inventory-II [26]).

Individuals eligible for study participation received a link to the baseline assessment. After completing the baseline assessment and giving informed consent for study participation, participants were randomly assigned to one of the two study groups using a computer-generated randomization list with a ratio of 2:2 and a block size of four. The list was generated by a member of the department of the first and last author, who was not otherwise involved in this study. Randomization was conducted anonymously, without any personal contact between study personnel and participants. Blinding of participants was infeasible because participants were aware of whether they were randomized to the IG or the WL. Participants were informed about their group allocation via email and, depending on the randomization outcome, either were granted immediate access to the web- and app-based intervention (IG) or were promised access after 3 months of waiting time (WL).

### Intervention

The feasibility and preliminary effectiveness of the web- and app-based resilience intervention RESIST were positively evaluated in a pilot RCT [17]. As a genuine resilience framework, PASTOR was considered the underlying theoretical foundation of the intervention [2,17]. Overall, the intervention aims to promote a positive appraisal style of stressors and stressful situations to help users adjust to work-related stress. Thus, this study design followed an approach focused on delivering the intervention during ongoing stressor exposure [12]. According to PASTOR, both a positive appraisal style as a resilience mechanism and successful adaptation to stress are facilitated by several resilience factors. Thus, promoting a set of specific resilience factors was at the intervention’s core. However, PASTOR is a conceptual framework for this study of resilience, aiming for comprehensiveness in explaining the mechanisms underlying resilient adaptation. Consequently, it does not provide specific guidelines on which factors should be strengthened or how this should be achieved. Thus, the selection of the resilience factors targeted in the training was guided by several considerations: (1) they should be etiologically relevant for fostering adaptive responses to adversity; (2) they should potentially be competencies fundamental to resilience; (3) they should encompass different facets of resilience, including behavioral (eg, self-efficacy), cognitive (eg, optimism), emotional (eg, self-compassion), and interpersonal (eg, social support) dimensions; (4) they should reflect varying temporal orientations (eg, self-efficacy grounded in past achievements and optimism oriented toward the future); (5) they should be distinct from factors commonly targeted in other well-established intervention approaches (eg, mindfulness or active coping, which are typically addressed in the context of mindfulness and stress-management training); and (6) the number of selected factors should remain manageable to enable the design of medium-length interventions, which have been shown to be most effective [27]. Based on these considerations, self-efficacy, optimism, self-compassion, and social support [28-31] were selected as resilience factors targeted in the training.

RESIST was designed as a hybrid intervention to be delivered individually to participants, combining a web-based intervention and a mobile app component. The intervention’s format allows participants to complete it anywhere they prefer. The web-based component accessible via a digital platform [32], consisting of longer intervention sessions, is designed for completion on a one-per-week basis. Once one session is completed, the next session is unlocked automatically. The app component that can be downloaded from the Google Play Store and Apple App Store (no longer available) consists of daily exercises. The two components are intentionally interrelated, with daily app exercises informing web-based sessions and web sessions guiding app-based practice. The intervention period for each study participant was 8 weeks. A detailed overview of the interventions’ content and exercises can be found in Multimedia Appendix 2. Strengths-Based CBT [23] is applied as the interventional technique addressing *how* the resilience factors are targeted and is comprised of four steps: (1) searching for strengths; (2) building a personal model of resilience (PMRe), that is, a positive self-concept including positive imagery or metaphors; (3) applying the PMRe; and (4) practicing the PMRe.

Using the app component was step 1 (search for strengths) of the Strengths-Based CBT approach (Multimedia Appendix 2). As the app’s central feature, users can document experiences of resilience, so-called moments of resilience, throughout the day (see Figure S2A in Multimedia Appendix 3). These moments capture instances when users overcome minor or major obstacles or experience positive events despite ongoing stressors or challenging life circumstances. During a daily review, these moments are categorized into one of the four resilience factors of self-efficacy, optimism, self-compassion, and social support, based on the user’s descriptions (see Figure S2D in Multimedia Appendix 3). Details on other app components are provided in Multimedia Appendix 3.

The web-based component has six sessions. The first session introduces the resilience intervention. Each of the second through fifth sessions addresses step 2 (construct PMRe), step 3, and step 4 (apply+practice PMRe) of the Strengths-Based CBT approach (Multimedia Appendix 2), which are incorporated as follows. The moments of resilience collected with the app are displayed in the web component. Based on step 2 of the Strengths-Based CBT approach, an exercise named resilience self-image was designed to further work with the moments of resilience. Within this exercise, users can select one of the displayed moments of resilience and develop a positive imagery or metaphor describing how they felt in the respective moment (eg, “I felt like a tribal leader, aware of my position and bravely taking responsibility”). This was intended to help participants develop a positive and resilient self-concept. A third exercise, named the resilience project, was developed based on steps 3 and 4 (apply+practice PMRe) of the Strengths-Based CBT approach. Within this exercise, users can plan how to approach and overcome an upcoming stressful situation by making use of their resilience self-image and resilience factors. The sixth session recaps the content of all the previous sessions.

Besides the exercises constructed on the Strengths-Based CBT, sessions include educational elements via text or video, as well as further written or audio exercises on the four resilience factors, each targeted in a single session. The sessions further feature two personas, which represent fictional users who are also completing the resilience training, thereby providing examples of how to complete the exercises. Example screenshots of the web component are available in Multimedia Appendix 3. Each session consists of 8‐10 web pages and requires 45‐60 minutes to complete. Users may complete the intervention at their own pace; however, it is recommended that they finish one session per week. No external reminders were sent to participants to complete the web-based component; however, in-app reminders could be set within the app component. The intervention was not tailored or modified during the course of this study. Access to the intervention can be provided to readers upon request.

### Measures

#### Overview

Web-based data assessment via patient-reported outcome measures took place before randomization for screening purposes (T0), at baseline (T1), immediately postintervention, which was eight weeks after randomization (postintervention assessment [T2]), and three months after randomization (3 mo follow-up [T3]). We further included a 6-month follow-up (T4) assessment for the IG only, guided by existing evidence indicating that between-group intervention effects of similar programs remain largely stable over this period [10,33]. Thus, for ethical reasons, the control group did not continue without intervention beyond the initial 3 months.

#### Primary Outcome Measure

The primary outcome of this study was subjective stress referring to the last 7 days, measured using the Perceived Stress Scale-10 (PSS-10) [34,35], a well-established self-report measurement that assesses the degree to which people appraise situations in life as stressful—that is, unpredictable, uncontrollable, or overloaded. It consists of 10 items (eg, “In the last month, how often have you felt nervous and ‘stressed’?”) with each item rated on a scale ranging from 0 to 4. The total score ranges from 0 to 40, with higher scores indicating higher levels of perceived stress. In the present sample, the scale demonstrated high internal consistency, with an *α*=.89 at baseline.

#### Secondary Outcome Measures

##### Overview

Secondary explorative outcomes included resilience-, mental health-, work-, and intervention-related outcomes. Further details and the reliability of all outcome measures are listed in Multimedia Appendix 4.

##### Resilience and Resilience Factors

Self-perceived resilience—the perceived ability to recover from stress—was measured using the Brief Resilience Scale [36]. The resilience factor self-efficacy was assessed using the General Self-Efficacy Short Scale-3 [37]. As related constructs, internal and external control beliefs were estimated using the Short Scale for the Assessment of Locus of Control [37] for explorative purposes. The resilience factor, optimism, was assessed using the revised version of the Life Orientation Test [38]. The resilience factor, self-compassion, was assessed using the Self-Compassion Scale Short Form [39]. The resilience factor, social support*,* was measured with the perceived available social support subscale and the support-seeking subscale of the Berlin Social Support Scales [40].

##### Mental Health Outcomes

Symptoms of depression were measured using the Epidemiologic Studies Depression Scale [41]. Values ≥18 indicate clinically significant levels of depressive symptoms [42]. Psychological distress, including anxiety and psychosomatic symptoms, was measured using the short version of the Brief Symptom Inventory [43].

Consistent with the notion that resilience per se is defined as good mental health despite adversity [44], exposure to different types of stressors was assessed. Critical life events (eg, death of a family member) were measured using an adapted version of the Life History Calendar [45]. Daily hassles were assessed using the Mainz Inventory of Microstressors [46].

##### Work-Related Outcomes

Adverse circumstances at work were assessed using the short version of the Effort-Reward-Imbalance Questionnaire [47,48], which contains the subscales effort, reward, and overcommitment. For the purpose of describing this study’s sample baseline characteristics, the ratio of effort (numerator) over reward (denominator) at T1 was calculated to capture the imbalance between costs and gains experienced at work [49]. According to Lehr et al [49], a ratio >0.715 indicates an adversarial workplace environment.

Concerning productivity in the workplace, days of absenteeism and presenteeism were measured using the respective items of the Trimbos and Institute of Medical Technology Assessment Cost Questionnaire for Psychiatry [50]. Ability to work effectively was measured using the single-item Work Ability Index [51], assessing “current work ability compared with life-time best.”

Due to data management errors, data on the secondary outcomes, psychological distress, effort, and rewards are missing for T2 and T4, while data for life events and daily hassles are missing from T2 to T4. These missing data represent minor deviations from this study’s protocol.

##### Intervention Usage and Client Satisfaction

Intervention usage was assessed in two ways: (1) by the number of web-based sessions completed per IG participant (extracted from the online platform); and (2) by the collected number of moments of resilience, the core feature of the app, per participant (as tracked by the native application). Satisfaction with the web- and app-based intervention was assessed postintervention (only for IG) using an adapted version of the Client Satisfaction Questionnaire adjusted to the online format of the intervention [52]. Participants’ perception of the app’s attractiveness and quality was assessed using the short version of the AttrakDiff [53].

To characterize this study’s sample, data on demographic variables such as age, gender, marital status, educational level, employment status, income, and the previous use of health services were collected as part of the screening procedure.

### Statistical Analysis

#### Overview

In accordance with CONSORT-eHEALTH guidelines [24], data were analyzed following an intention-to-treat procedure. Specific details of the analysis are provided below. All statistical analysis was performed using R Studio (R version 4.3.2) [54] with a two-tailed significance level set at *P*≤.05.

#### Power Calculation

This study’s sample size calculation was based on identifying a realistic and practically meaningful effect size. First, regarding a realistically achievable effect, meta-analytic evidence on unguided web-based interventions for stress reduction was considered [27]. In the meta-analysis by Heber et al [27], an effect size of *d*=−0.33 was reported for unguided stress interventions. Second, by discussion, the research team agreed on 2.5 points as the minimal practically important difference between the IG and WL immediately after the intervention. Assuming an SD of 6.42 points on the PSS-10, as observed in a representative German sample [34], this resulted in an anticipated intervention effect of *d*=−0.4 for the primary outcome in this study. To detect an effect of that size with 80% power and 95% significance, an a priori power-analysis calculation yielded 352 participants required for this study.

#### Missing Data

All participants provided baseline data. Missing data at T2-T4 were imputed through multivariate imputation by chained equations using the *mice* package [55]. Twenty imputation sets were generated. Variables were included as predictors in the imputation model if they showed a correlation of at least 0.4 with the variable to be imputed and had at least 60% available data, ensuring that only predictors carrying a substantial amount of meaningful information were used [56]. The imputed datasets were analyzed separately, and the parameter estimates and hypothesis tests were ultimately pooled using the rule by Rubin [57].

#### Intervention Effect

Between-group differences at T2 and T3 were analyzed using analysis of covariance (ANCOVA), as recommended by O’Connell et al [58], to evaluate the interventions’ effectiveness in influencing the primary and secondary outcomes. Baseline scores of the respective outcomes were considered covariates. Following recommendations by Harrer et al [56], between-group Cohen *d* and corresponding CIs were derived directly from the covariate-adjusted models used to calculate the magnitude of treatment effect, incorporating the pooled SD (based on Cohen formula) into the models.

Sensitivity analyses for the primary outcome were performed to test the robustness of the intention-to-treat analyses in multiple ways. First, study completers (participants providing data at all measurement points) were analyzed. Second, intervention completers were analyzed, defined as participants having completed at least the first five sessions of the web-based component (incorporating all exercises on the targeted four resilience factors) and collected moments of resilience with the app at least twice per week (≥16) within the training period. Third, the intervention effect was measured using linear mixed models.

#### Response and Deterioration Rates

Concerning better communicating the results to help-seekers and policy makers, we followed the recommendation by Cuijpers [59] and calculated response and deterioration rates. For all response rates, we calculated the numbers needed to treat to achieve one additional beneficial outcome (number needed to treat for benefit; NNTB) and one additional harmful outcome (numbers needed to treat for harm; NNTH) in the IG, relative to the WL.

We analyzed response rates from various perspectives. First, reliable improvement and deterioration were calculated based on Heber et al [33], who reported a change of ±5.16 points in the PSS-10 following the recommendations of Jacobson and Truax [60]. Second, using anchor-based criteria was advised for determining what constitutes a practically meaningful change [61]. Bauer-Staeb et al [62] highlighted that the magnitude of a practically meaningful change depends on the baseline level of distress and provided reference criteria. Given that the sample was expected to be primarily mildly to moderately distressed, a change of 20% from baseline to postintervention was considered a minimally practical meaningful difference. Third, remission was determined using the preferred method described by Jacobson and Truax [60] for operationalizing clinically significant change—defined as having a score closer to the mean of the functional than dysfunctional population. The mean for a representative German sample (mean 12.57) served as an estimate for the functional population [34], while the mean score of a highly stressed, help-seeking sample (mean 22.65) was used to define the dysfunctional population [63]. Consequently, we defined remission as scoring 17 points or lower on the PSS-10, excluding participants already considered to have no significant symptoms by this definition at baseline.

#### Longer-Term Effects

An extended T4 assessment evaluated longer-term interventional effects among participants in the IG. Those effects were analyzed using within-subject comparisons via repeated-measures ANOVA between T1 and T4. Within-group Cohen *d* and respective CIs for the IG were calculated by dividing the change score (T1-T4 difference) by the SD of the change.

#### Mediation Analyses

To investigate the targeted resilience factors self-efficacy, optimism, perceived support, and self-compassion as potential mediators of the intervention’s effect on stress and resilience, multiple single (including one mediator) and parallel mediation analyses (including all mediators at once) were conducted using the *semTools* package [64]. Single mediation analyses were conducted to assess the unique effect of each mediator in isolation, while parallel models provided a more comprehensive view of how multiple mediators operate concurrently and allowed for comparison of their relative contributions. To establish temporal precedence, T2 scores of the potential mediators and T3 scores of stress and resilience were used [18]. Mediator and baseline scores for stress and self-perceived resilience were included as covariates in the model, as recommended by Hayes and Rockwood [65]. A statistically significant effect of the mediation was achieved if the estimated 95% CI of the indirect effect did not cross zero. We conducted all mediation analyses with the study completer sample as additional sensitivity analyses.

## Results

### Participants and Baseline Characteristics

**Figure 1.**
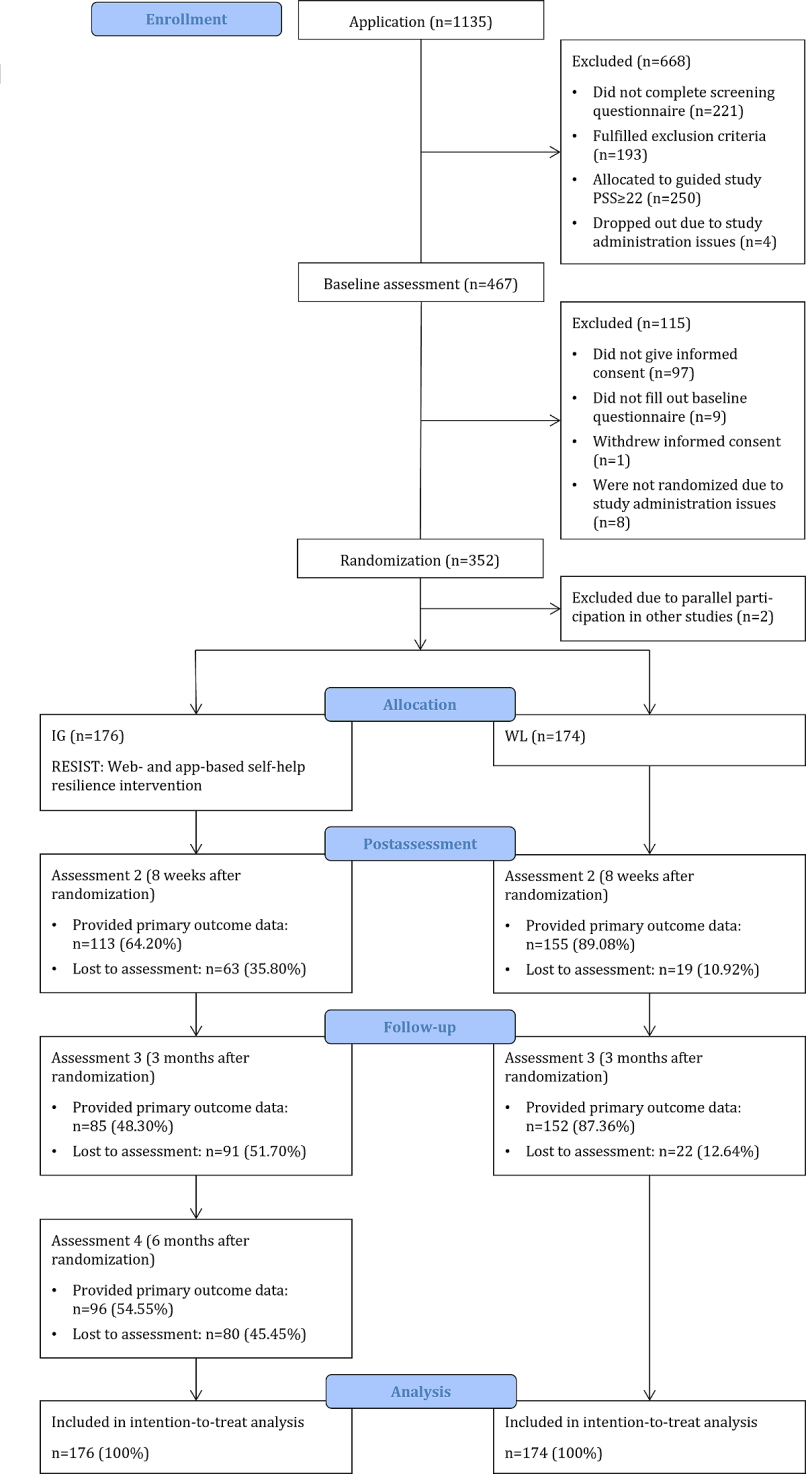
Study flow of participants. IG: intervention group; PSS: Perceived Stress Scale; WL: waitlist control group.

Figure 1 illustrates the flow of participants. A total of 352 individuals were randomized and assigned to either the IG or WL. Two participants initially allocated to the WL were subsequently excluded from this study due to concurrent involvement in another parallel research project. As a result, the final participant count was 176 participants for the IG and 174 participants for the WL.

Table 1 summarizes the study samples’ baseline characteristics. On average, participants were aged 42.8 years; 65.7% (230/350) were female; and 74.9% (262/350) worked full-time. At screening, participants reported an average perceived stress level of 21.58 (SD 6.03). The average depression level among participants at baseline was 11.09 (SD 6.42). A total of 15.7% (55/350) of the participants reported clinically relevant levels of depressive symptoms. Participants reported an average sum of 68.45 (SD 32.72) daily hassles in the preceding 7 days (eg, a conflict or disagreement with a close person), along with 9.24 (SD 5.08) experienced life events (eg, serious illness of self or family member). Almost nine in ten (309/350, 88.3%) reported working under adversarial workplace conditions (Effort Reward Imbalance Scale ratio >0.715) at baseline. Participants’ work ability—compared to life-time best indicated as 10 points—was reduced (mean 6.83, SD 1.86).

**Table 1.** Baseline characteristics of this study’s sample.

	Total	IG^a^ (n=176)	WL^b^ (n=174)
Sociodemographics			
Age (y), mean (SD)	42.8 (11.2)	41.9 (11.2)	43.8 (11.1)
Female, n (%)	230 (65.7)	123 (69.9)	107 (61.5)
Single, n (%)	116 (33.1)	67 (38.1)	49 (28.2)
Married, n (%)	169 (48.3)	77 (43.8)	92 (52.9)
Relationship, n (%)	40 (11.4)	18 (10.2)	22 (12.6)
Divorced, n (%)	25 (7.1)	14 (8)	11 (6.3)
Education, n (%)			
No university degree	81 (23.1)	41 (23.3)	40 (23)
University degree	269 (76.9)	135 (76.7)	134 (77)
Employment, n (%)			
Full-time	262 (74.9)	143 (81.3)	119 (68.4)
Part-time	88 (25.1)	33 (18.8)	55 (31.6)
Prior experience with a mental health intervention, n (%)			
No	310 (88.6)	152 (86.4)	158 (90.8)
Yes	40 (11.4)	24 (13.6)	16 (9.2)
Experience with psychotherapy, n (%)			
No, never	243 (69.4)	123 (69.9)	120 (69)
Yes, in the past	103 (29.4)	52 (29.6)	51 (29.3)
Perceived stress levels, mean (SD)			
T0 PSS-10^c^	21.6 (6.03)	21.8 (5.7)	21.4 (6.4)
Clinically significant levels of depressive symptoms^d^, n (%)			
Yes	55 (15.7)	22 (12.5)	33 (19)
No	295 (84.3)	154 (87.5)	141 (81)
Stressor exposure before intervention			
Adverse workplace situation (ERI-S^e^>0.715), n (%)	309 (88.3)	153 (86.9)	156 (89.7)
Macrostressors^f^, mean (SD)	9.24 (5.1)	9.1 (4.9)	9.4 (5.2)
Microstressors^g^, mean (SD)	68.5 (32.7)	68.1 (32.2)	68.8 (33.3)

a IG: intervention group.

b WL: waitlist control group.

c PSS-10: Perceived Stress Scale 10.

d Scores ≥18 on the Epidemiologic Studies Depression Scale (short form).

e ERI-S: Effort-Reward-Imbalance Scale (short form).

f Life events (counts).

g Daily hassles (counts).

### Primary Outcome Measure

Table 2 lists the means and SDs of the primary and all secondary outcomes at all assessment points. At T2, individuals in the IG reported significantly lower levels of perceived stress than individuals in the WL, as demonstrated by ANCOVA (*F*_1,518_=21.31, *P*<.001, *d*=−0.54, 95% CI −0.75 to −0.34, between-group difference: −3.14 points, see Table 3). The between-group effect remained significant and comparable in size at 3-month follow-up (*F_1,79_*=10.38, *P*=.002, *d*=−0.47, 95% CI −0.71 to −0.22, between-group difference: −2.79, see Table 3).

**Table 2.** Means and SDs of the intention-to-treat approach’s sample (intervention group=176; waitlist control group=174).

Outcome		T1^a^		T2^b^		T3^c^		T4^d^
		IG^e^	WL^f^	IG	WL	IG	WL	IG
Primary outcome, mean (SD)								
	Stress	21.35 (6.21)	20.52 (6.71)	16.22 (6.47)	19.36 (6.63)	16.63 (7.33)	19.42 (6.92)	16.11 (6.86)
Secondary outcomes, mean (SD)								
	Self-perceived resilience	16.80 (4.70)	16.61 (4.44)	18.84 (3.40)	17.19 (3.31)	19.34 (4.88)	17.86 (4.36)	19.67 (4.48)
	Resilience factors, mean (SD)							
	Self-efficacy	10.52 (2.13)	10.30 (2.23)	11.25 (2.22)	10.85 (2.02)	11.20 (2.25)	10.62 (2.01)	11.56 (1.98)
	Internal control	3.58 (0.76)	3.50 (0.75)	3.81 (0.69)	3.56 (0.75)	3.70 (0.83)	3.46 (0.77)	3.77 (0.79)
	External control	2.72 (0.06)	2.65 (0.80)	2.40 (0.80)	2.63 (0.77)	2.33 (0.96)	2.61 (1.05)	2.32 (0.70)
	Optimism	14.06 (4.63)	13.90 (4.48)	15.59 (4.62)	14.46 (4.44)	15.89 (4.86)	14.35 (4.72)	16.11 (4.37)
	Self-compassion	2.75 (0.72)	2.76 (0.68)	3.27 (0.71)	2.87 (0.69)	3.19 (0.73)	2.87 (0.69)	3.25 (0.74)
	Perceived support	26.90 (4.83)	26.16 (5.20)	27.92 (4.38)	26.96 (4.89)	27.63 (4.71)	26.68 (4.90)	27.99 (4.70)
	Support seeking	13.48 (3.84)	13.40 (3.76)	14.55 (3.60)	13.62 (3.54)	14.58 (3.70)	13.39 (3.47)	14.75 (3.42)
	Mental health, mean (SD)							
	Depressive symptoms	10.85 (6.19)	11.33 (6.64)	9.61 (6.16)	11.41 (6.41)	9.61 (6.16)	11.12 (6.61)	9.68 (6.06)
	Psychological distress	0.83 (0.53)	0.82 (0.55)	—^g^	—	0.68 (0.52)	0.74 (0.51)	—
	Work-related health, mean (SD)							
	Work ability	6.80 (1.99)	6.86 (1.73)	7.26 (1.98)	6.75 (1.90)	6.73 (2.27)	6.84 (1.85)	7.17 (2.15)
	Effort	8.61 (2.30)	8.83 (2.11)	—	—	7.82 (2.47)	8.59 (2.35)	—
	Reward	18.79 (3.75)	18.02 (3.93)	—	—	18.74 (3.92)	17.96 (4.04)	—
	Over-commitment	15.99 (3.73)	16.06 (3.44)	14.74 (3.68)	15.61 (3.69)	14.33 (3.55)	15.69 (3.77)	14.28 (3.78)
	Absenteeism	7.01 (7.75)	8.05 (11.35)	5.80 (7.88)	8.20 (12.44)	3.69 (4.48)	4.63 (5.75)	7.34 (9.29)
	Presenteeism	6.34 (4.90)	7.32 (9.05)	6.53 (6.30)	6.67 (5.67)	6.66 (6.62)	5.26 (4.17)	4.08 (2.89)

a T1: baseline.

b T2: postintervention (8 weeks after randomization).

c T3: 3-month follow-up (3 months after randomization).

d T4: 6-month follow-up (6 months after randomization, intervention group only).

e IG: intervention group.

f WL: waitlist control group.

g Missing due to data management errors.

**Table 3.** Between-group differences postintervention and at 3-month follow-up for primary and resilience-related secondary outcome measures.

Outcome		Differences between the intervention group and the waitlist control group					
		T2^a^			T3^b^		
		*F* test (*df*)	*P* value	Cohen *d* (95% CI)	*F* test (*df*)	*P* value	Cohen *d* (95% CI)
Primary outcome							
	Stress	21.31 (1, 518)	<.001	−0.54 (−0.75 to −0.34)	10.38 (1, 79)	.002	−0.47 (−0.71 to −0.22)
Secondary outcomes							
	Self-perceived resilience	30.46 (1, 253)	<.001	0.47 (0.29 to 0.64)	9.33 (1, 72)	.003	0.29 (0.08 to 0.49)
	Resilience factors						
	Self-efficacy	4.15 (1, 248)	.04	0.12 (0.06 to 0.30)	4.87 (1, 69)	.03	0.21 (−0.03 to 0.45)
	Internal control	11.23 (1, 191)	<.001	0.27 (0.06 to 0.47)	7.82 (1, 181)	.006	0.24 (0.03 to 0.45)
	External control	6.82 (1, 132)	.01	−0.35 (−0.57 to −0.12)	4.76 (1, 81)	.03	−0.33 (−0.57 to −0.08)
	Optimism	6.35 (1, 49)	.02	0.22 (0.16 to 0.60)	12.38 (1, 89)	.001	0.30 (0.11 to 0.48)
	Self-compassion	36.23 (1, 106)	<.001	0.57 (0.39 to 0.76)	11.36 (1, 46)	.002	0.42 (0.18 to 0.66)
	Perceived support	5.09 (1, 74)	.03	0.09 (−0.09 to 0.27)	4.18 (1, 78)	.04	0.09 (−0.10 to 0.27)
	Support seeking	11.92 (1, 380)	.001	0.25 (−0.09 to 0.40)	12.89 (1, 92)	.001	0.32 (0.14 to 0.49)

a T2: postintervention (8 weeks after randomization).

b T3: 3-month follow-up (3 months after randomization).

### Sensitivity Analyses

Sensitivity analysis based on study completers (T2: *d*=−0.64, 95% CI −0.89 to −0.39; T3: *d*=−0.55, 95% CI −0.82 to −0.28) corroborated the results obtained by intention-to-treat analysis. The group difference between intervention completers and the WL at T2, however, was only close to being statistically significant (*P*=.05, *d*=−0.42, 95% CI −0.79 to −0.06). In contrast, the group difference at T3 was significant and similar in size to that obtained by the intention-to-treat approach (*d*=−0.46, 95% CI −0.84 to −0.09). A linear mixed-effects model revealed results similar to those obtained by ANCOVA using imputed data. Equivalently, the interactions between time and intervention at T2 (*P*<.001, SE=0.75) and T3 (*P*<.001, SE=0.80) were significant, indicating that the change in stress differed between groups. Specifically, participants in the IG showed a significantly greater reduction in stress from T1 to T2 (*β*=−4.02, 95% CI −5.48 to −2.56), as well as from T1 to T3 (*β*=−3.67, 95% CI −5.24 to −2.11), compared to those on the WL.

### Response and Deterioration Rates

At T2 and T3, significantly more participants in the IG than WL controls reported reliable and practically meaningful improvement, with NNTB ranging from 3.42 (95% CI 2.56 to 5.16) to 4.35 (95% CI 3.05 to 7.58). The rates of reliable and practically meaningful deterioration were higher in controls than in the IG at both postintervention points, with NNTH values ranging from 7.51 (95% CI 4.80 to 17.21) to 10.81 (95% CI 6.57 to 30.57). However, 4.6% (8/174) to 10.9% (19/174) of participants in the IG also experienced a worsening of stress symptoms. Multimedia Appendix 5 summarizes the results of the response and deterioration analysis.

### Secondary Outcome Measures

Table 3 summarizes the results of resilience-related secondary outcome analysis. For self-perceived resilience, there was a significant effect in favor of active treatment at T2 (*d*=0.47) and T3 (*d*=0.29). For the resilience factors, there were significantly better outcomes among participants in the IG for all outcomes at T2 and T3. Immediately postintervention, effect sizes were 0.12 for self-efficacy, 0.27 for internal control, −0.35 for external control, 0.22 for optimism, 0.57 for self-compassion, 0.09 for perceived social support, and 0.25 for support seeking.

Multimedia Appendix 6 summarizes the findings of the other mental health- and work-related outcomes assessed. For depression, there was a significant between-group effect at T2 (*d*=−0.24), but not at T3. Regarding work-related outcomes, significant effects in favor of the IG were found for work ability at T2 (*d*=0.28), and for overcommitment at T2 (*d*=−0.22) and T3 (*d*=−0.36), but not for absenteeism or presenteeism at either of these postintervention measurement points.

### Longer-Term Effects

Concerning the intervention’s long-term effectiveness (T1-T4), outcomes remained stable, indicating that the beneficial effects were maintained. Within-group effect sizes for the primary and secondary outcomes were all significant, except for absenteeism. The effect size for stress was *d*=−0.80 (95% CI −0.97 to −0.63), for self-perceived resilience *d*=1.19, for self-efficacy *d*=0.80, for optimism *d*=0.87, for self-compassion *d*=1.25, and for perceived social support *d*=0.37. A table summarizing the results of the within-group comparisons obtained by repeated-measures ANOVA is available in Multimedia Appendix 7.

### Mediation Analyses

#### Perceived Stress

Single mediation analysis revealed that indirect effects through optimism (ab=−0.46, 95% CI −0.80 to −0.12) and self-compassion (ab=−0.95, 95% CI −1.49 to −.41) were statistically significant, indicating that these variables mediated the effect of the intervention on perceived stress at T3. In contrast, indirect effects through self-efficacy (ab=−0.11; 95% CI −0.34 to 0.12) and perceived support (ab=−0.04; 95% CI −0.18 to 0.10) were not significant.

**Figure 2.**
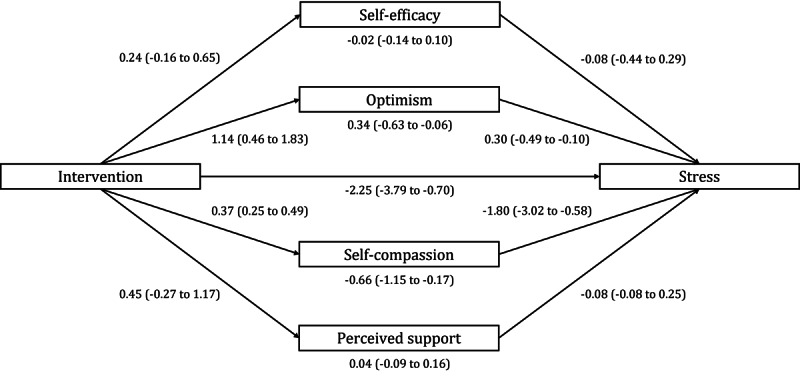
Parallel multiple mediation model with 3-month follow-up (T3) stress severity scores as the outcome variable (Y), posttreatment (T2) resilience factors scores as mediators, and baseline values of mediators and outcome as covariates. Intervention (X) is coded 1=IG, 0=WL. Unstandardized beta coefficients are shown with 95% CIs in brackets. IG: intervention group; WL: waitlist control group.

As depicted in Figure 2, the results of parallel mediation analysis aligned with the results obtained with single mediation analyses. In a joint model, the indirect effects through optimism (a_2_b_2_=−0.34, 95% CI −0.63 to −0.06) and self-compassion (a_4_b_4_=−0.66, 95% CI −1.15 to −0.17) were again statistically significant, while the indirect effects through self-efficacy (a_1_b_1_=−0.02, 95% CI −.14 to 0.10) and perceived support (a_3_b_3_=0.04, 95% CI −0.09 to 0.16) were not. The direct effect of the intervention on perceived stress remained significant after incorporating the mediators in the model (*c*’=−2.25, 95% CI −3.79 to −0.70).

#### Self-Perceived Resilience

Mediation analyses with self-perceived resilience—that is, the ability to recover from stress—as a dependent outcome revealed a pattern similar to that observed with perceived stress as the outcome. Single mediation analyses showed that the indirect effects through optimism (ab=0.27, 95% CI 0.07 to 0.46) and self-compassion (ab=0.46, 95% CI 0.16 to 0.76) were statistically significant, indicating that these variables mediated the intervention’s effect on self-perceived resilience at T3. In contrast, indirect effects through self-efficacy (ab=0.08; 95% CI −0.06 to 0.22) and perceived support (ab=0.01; 95% CI −0.07 to 0.08) were not.

On parallel mediation analysis, the indirect effect through optimism (a_2_b_2_=0.21, 95% CI 0.06 to 0.37) significantly mediated the intervention’s effect on resilience at T3, while the indirect effect through self-compassion (a_4_b_4_=0.26, 95% CI −0.01 to 0.53), though close, failed to achieve statistical significance. The indirect effects achieved through self-efficacy (a_1_b_1_=0.04, 95% CI −0.03 to 0.11) and perceived support (a_3_b_3_=−0.03, 95% CI −0.10 to 0.04) were also not statistically significant. The direct effect of the intervention on self-perceived resilience remained significant after incorporating mediators into the model (*c’*=0.86, 95% CI 0.04 to 1.65). The corresponding mediation model is shown in Figure S1 in Multimedia Appendix 8.

Repeating all single and parallel mediation analyses with this study completer sample as additional sensitivity analyses revealed no differences in the results relative to those obtained with the intention-to-treat approach (see Figures S2 and S3 in Multimedia Appendix 8). The only exception was in the joint model with self-perceived resilience as the dependent variable, in which the indirect effect via self-compassion reached statistical significance (a_4_b_4_=0.43, 95% CI 0.13 to 0.73).

### Intervention Usage and Client Satisfaction

Most participants started with the web component of the intervention (156/176, 88.6%), completing an average of 2.2 (SD 2.3) sessions. Completion rates ranged from 70.5% (110/156) for session 1 to 18.6% (29/156) for all 6 sessions.

Those who used the app component (104/176, 59.1%) collected a median of 14 moments of resilience (range 1 to 220), indicating that half collected these moments almost twice per week over the 8-week intervention period. Based upon participant reports, users collected a total of 3064 moments of resilience, most frequently for self-compassion. Detailed engagement patterns are summarized in Multimedia Appendix 9. Of those participants reporting their intervention usage at T2, 38.1% (32/84) indicated they had not yet finished and expressed an intention to continue.

Participants’ satisfaction with the intervention (considering both the web and app components) was high (mean 24.02, SD 6.18). Of those participants providing questionnaire data about their satisfaction with the intervention, 87/99 (87.9%) indicated satisfaction in an “overall, general sense” (“very satisfied” or “mostly satisfied”), and 80/99 (80.5%) indicated satisfaction with the amount of help they received. In addition, 73/99 (73.7%) agreed that the intervention helped them to deal with problems more effectively, and 77/99 (77.8%) said they would recommend the intervention to a friend in need of similar help. The overall user experience of the app was rated as 4.71 (SD 1.04; range 1 to 7; n=99), indicating an overall good level of user experience [53].

## Discussion

### Principal Results

#### Overview

For the first time, this study identified a favorable effect of the RESIST intervention, relative to being on a waiting list, on perceived stress in a universal prevention setting. Improvements were evident immediately after the intervention and remained stable 3 and 6 months after randomization. A similar pattern was observed for self-perceived resilience and the resilience factors targeted within the intervention. Effects on other health- and work-related outcomes were mixed.

Concerning the second study aim, mediation analyses suggested that the resilience factors targeted within the training played distinct roles in how the intervention exerted its effects on stress and self-perceived resilience. The strongest evidence for mediation emerged for optimism and self-compassion, with significant indirect effects observed for both variables. In contrast, indirect effects through self-efficacy and social support were not statistically significant.

#### Effects on Stress and Self-Perceived Resilience

The observed effect on stress reduction immediately postintervention (*d*=−0.54) slightly exceeded the effect size expected from the a priori sample size calculation (*d*=−0.40). Building on the findings from the pilot study [17], this study confirmed the intervention’s effectiveness in a larger sample within a universal preventive setting, demonstrating that the intervention is effective not only when delivered with guidance, as in the pilot study, but also in a self-help format that requires fewer resources.

When compared with interventions targeting a similar set of resilience factors that may be unique to resilience interventions [21,66], the effect sizes for stress and self-perceived resilience (*d*=0.47) observed in this study fall between those reported for a web-based resilience intervention for college students [66] (*d*=−0.34 for stress) and a multicomponent positive psychology intervention for the general population [21] (*d*=0.67 for resilience).

Concerning intervention techniques, this study’s findings align with those of 2 previous studies that also used Strengths-Based CBT [67,68]. In one of these studies [68], distressed college students showed significant postintervention improvements in resilience (*d*=0.34).

Compared to meta-analytic findings, the stress reduction effect observed for the present intervention postassessment was substantially larger than the nonsignificant effect (*g*=0.14) reported by Ang et al [7], who used a broad resilience factor approach. In contrast, the findings related to self-perceived resilience closely align with those of Ang et al [7] (*g*=0.54). The effect of RESIST on self-perceived resilience is also similar in magnitude to that reported for the meta-analysis by Díaz-García et al [8], which adopted an outcome-focused resilience approach, including interventions aimed at modifying resilience or related constructs regardless of the interventions’ characteristics. Furthermore, the stress reduction effect of RESIST was slightly larger than the effect size for stress reported in the meta-analysis by Schäfer et al [11] (standard mean difference=0.33, 95% CI −0.41 to −0.24), which applied a broad conceptualization of resilience interventions, also including established intervention formats such as stress-management and mindfulness programs. The effects for self-perceived resilience found in the present study also surpassed those reported by Schäfer et al [11] (standard mean difference*=*0.22).

Although these comparisons do not establish superiority of interventions that target a specific set of resilience factors that may reflect more overarching resilience processes and may be correlates of higher-order resilience mechanisms, such as a positive appraisal style, the findings suggest that it is valuable to continue developing and investigating interventions based on these principles. This is particularly important if resilience interventions are to be seen as a distinct form of intervention.

The comparisons further underscore the challenges of adequately evaluating and comparing resilience interventions, stemming from the lack of a clear understanding of what constitutes a resilience intervention. The variability in the design of resilience interventions makes it difficult to identify comparable interventions, especially when investigators fail to adequately describe the intervention’s characteristics. As mentioned earlier, such conceptual ambiguity is reflected in meta-analyses, where differing definitions of resilience interventions can lead to substantially heterogeneous results and conclusions between meta-analyses. This emphasizes the need for a standardized classification system for resilience interventions. Building on prior considerations by Chmitorz et al [12], (1) the theory or rationale underlying an intervention, (2) its specific content, (3) the intervention techniques applied, and (4) the timing of the intervention relative to stressor exposure should be considered as dimensions for building a systematic classification system of resilience interventions. This may contribute to identifying the core elements of effective intervention designs.

#### Response and Deterioration Rates

The NNTB suggests that, on average, three to four individuals must be given access to RESIST for a single individual to experience 20% symptom improvement or reliable improvement in stress symptoms immediately after the intervention. This aligns with the results of other digital mental health interventions such as self-guided stress management training [69]. Corresponding to findings from prior research [70,71], it must be noted, however, that some individuals (12/174, 6.9%) in our IG met the criterion for reliable deterioration. This highlights safety issues within resilience interventions. While resilience interventions labeled positively might seem more appealing than, for example, stress management interventions, this does not guarantee symptom improvement, as some users may experience adverse effects. Potential risks may include heightened frustration or self-blame if participants do not meet perceived expectations of “being resilient” [72]. The positively framed concept of “resilience” may furthermore create implicit pressure to bounce back, potentially reinforcing feelings of inadequacy when setbacks persist despite effort. In the systematic review by Chmitorz et al [12], none of the 43 RCTs included in the analysis assessed adverse effects. Hence, the potential harms of so-called “positive” interventions remain largely overlooked [71], underscoring the need for more conceptual and empirical research in this area.

#### Effects on Resilience Factors

Besides examining RESIST’s effectiveness in reducing stress as a primary outcome and resilience as an important secondary outcome, we also investigated whether the intervention promoted the resilience factors of self-efficacy, optimism, self-compassion, and social support that the intervention was designed to target. The effects observed postintervention were favorable, yet variable in magnitude.

For self-efficacy, we observed a small but statistically significant between-group effect after the intervention (*d*=0.12). This is numerically similar to the effect reported by Schäfer et al [11] in their meta-analysis (*d*=0.01). Regarding optimism (ie, the general expectation that one’s own outcomes will be positive [73]), our findings indicated a more sizeable intervention effect (*d*=0.22), in line with results from meta-analyses by Schäfer et al [11] and Malouff and Schutte [74]. The most pronounced effect at T2 was observed for self-compassion (*d*=0.57; ie, a supportive attitude toward oneself in times of suffering [75]). This finding mirrors results reported by Schäfer et al [11], where self-compassion also showed the largest effect among the resilience factors examined. For perceived social support, we found small between-group differences at T2 (*d*=0.09), which also aligns with published meta-analyses [31]. Despite the statistically significant findings, the practical significance of these effects is difficult to interpret due to the lack of established criteria for meaningful change in resilience factors. Future research should investigate such criteria, following the methodological recommendations of Cook et al [76].

#### Mediation Analyses

The mediation analyses further revealed that RESIST not only favorably fostered optimism and self-compassion, but that these factors also contributed to how the intervention exerted its positive effects. With one exception—self-compassion mediated the effect on self-perceived resilience only in this study’s completer sample—the mediation results were consistent, irrespective of whether stress or self-perceived resilience was assessed as the outcome, underscoring that the same resilience factors may matter for both positive and negative mental health outcomes. Comparing these mediation findings to prior research results is limited by the scarcity of studies that have investigated the mechanisms of change underlying resilience interventions. Our mediation findings align with isolated prior findings of the previously mentioned multicomponent email-guided positive psychology intervention [21], for which both optimism and self-compassion also emerged as mediators of the intervention’s effects.

The findings shed light on the importance of optimism and self-compassion as potential key processes in resilience promotion. In the case of optimism, enhancing the belief that one’s own outcomes will be positive may both strengthen someone’s confidence that a currently stressful situation can improve over time and foster the perception that such situations are manageable and temporary, thereby reducing perceived stress and strengthening self-perceived resilience. This, in turn, might reflect a positive appraisal style of stressors. This finding aligns with a substantial body of prior research, including both meta-analyses and longitudinal studies, that have revealed the importance of optimism for positive mental health outcomes [30,77-80]. For instance, in a meta-analysis published by Gallagher et al [30], a negative association was identified between optimism and posttraumatic stress disorder. Meanwhile, Romswinkel et al [80] identified optimism both as a predictor of reduced depressive symptoms and as a mediator in the relationship between job stress and depression.

Regarding self-compassion, fostering a kinder and warmer attitude toward oneself during times of stress might have enabled IG participants to better care for themselves and respond with reduced self-criticism, particularly when facing stressors rooted in personal mistakes or feelings of inadequacy [75]. By promoting a more accepting and supportive internal response, the intervention may have helped them to reinterpret such stressors more constructively, thereby establishing a link to a positive appraisal style and ultimately reducing perceived stress. Empirical evidence supports this strong link between self-compassion and mental health [81-85]. For example, one meta-analysis has demonstrated an inverse association between self-compassion and stress [82], while a longitudinal study by Lee et al [81] identified self-compassion as a significant predictor of mental well-being over five years.

In contrast to self-compassion and optimism, no significant mediation effects were observed for self-efficacy and social support. However, it is premature to discount the roles of self-efficacy and social support in interventions aiming to promote resilience. Differences in the control and ease of implementation, as well as the timescale of observable change, may partly explain the pattern of effects. Self-compassion, for example, can be cultivated through self-directed practices, as evidenced by participants’ predominant selection of moments of resilience related to this factor, according to app data. In contrast, fostering self-efficacy could rely more on sufficient learning opportunities and external feedback [86], which may be provided by a mental health professional, such as an eCoach. Increases in perceived social support might depend more on social interactions and environmental factors, potentially requiring more time to become manifest. Including peer support in the intervention could serve as one valuable option to enhance these social interactions.

### Strengths and Limitations

This study has several key strengths that help it contribute to the body of research in the field. Its first strength is that it was conducted in accordance with the recommendations for resilience intervention studies outlined by Chmitorz et al [12], including an outcome-oriented resilience definition, and the separate assessment of various mental health outcomes and resilience factors, as well as adverse effects.

Second, to the best of our knowledge, the resilience intervention RESIST examined in this study is among the first to be explicitly grounded in a genuine resilience framework—namely, PASTOR [2], which served as the theoretical foundation of the intervention.

Third, the intervention’s content was informed by resilience factors considered etiologically relevant for fostering adaptive responses to adversity [87] and which may reflect resilience-specific competencies.

Fourth, the intervention specifically targeted resilience factors that are typically not addressed in established mental health programs, such as stress management or mindfulness-based interventions. Lastly, by studying mediators, this study addresses the call to study mechanisms of change to optimize intervention development and treatment outcomes from digital resilience interventions [11].

Despite this study’s strengths, the results should be interpreted in light of some limitations, with the first two relating to generalizability, the next two to methodological aspects, and the final two to theoretical considerations.

First, the findings may have limited generalizability to other implementation settings; for example, RESIST’s effectiveness could differ if the intervention is employer-provided rather than individually initiated. Employees may view employer-led resilience training as contradictory if it ignores structural causes of stress. This concern is supported by a meta-analysis that detected smaller-than-expected effects for organization-implemented e-mental health interventions [88].

Second, this study’s sample was predominantly female and highly educated, which may also limit generalizability. However, systematic reviews suggest that sociodemographic factors such as gender or education rarely modify intervention effects [89-91]. The main challenge may therefore be promoting uptake in other groups. Whether this may be achieved, for example, via approaches such as participatory design, tailored recruitment, or interventions addressing mental health indirectly (eg, physical exercise [92]), is not clear yet.

Third, the dropout rate across both intervention arms (82/350, 23.4%) could have negatively affected the results’ validity. However, this extent of attrition falls well within the range of similar digital self-help interventions [69,71], and the multiple imputations approach used to address this shortfall is both a robust and widely accepted method for handling missing data [93]. In addition, a range of sensitivity analyses, including mixed model analyses, supported the robustness of the intention-to-treat sample’s findings.

Fourth, nearly 40% (32/83) of participants indicated postintervention that they had not yet completed the intervention but intended to continue engaging with it. This may reflect the nature of the self-help format, which typically allows for greater flexibility. It also suggests that the intended pacing, one session and at least 2 app entries per week, may not have been well-matched to participants’ preferences or natural usage patterns.

Fifth**,** the applied mediation model implicitly assumes that all mediators change simultaneously over time, thereby limiting its capacity to account for temporal variability in their effects—some mediators may exert their influence more quickly, while others do so more slowly. This limitation is common in studies assessing such models.

Lastly, the interpretability of the mediation analyses’ results is limited with regard to drawing conclusions about any potential causal link between specific mediators and individual training components. For instance, it cannot be concluded that the content related to the resilience factors within the training necessarily encompasses the active ingredients driving the intervention’s effects. Component studies are needed to further investigate the active ingredients of RESIST.

### Theoretical Implications

Based on the study’s insights, various theoretical implications and perspectives for future research emerge. First, almost 90% (309/350) of the participants reported experiencing an effort-reward imbalance in their work life and, accordingly, trained resilience in the context of ongoing stressor exposure. A next step would be to more precisely assess the specific stressors experienced by participants and relate them to mental health outcomes, as proposed by Kalisch et al [94]. This would, for instance, enable meaningful comparisons between individuals who show similar improvements in mental health after training, despite facing different levels of adversity.

Second, while the mediation analyses provided initial and valuable insights into the intervention’s mechanisms of change, they also highlight several avenues for future research. The precise interrelations, causal directions, and underlying mechanisms linking the resilience factors optimism, self-compassion, and a positive appraisal style as potential resilience mechanisms warrant further investigation to deepen our understanding of their contribution to psychological adaptation and resilience. Further, for the investigation of RESIST, a logical next step will be to include an assessment of positive appraisal style [95] as an additional construct (which has been published just recently) in future assessments and to examine it as a potential mediator in sequential mediation, onto which the various resilience factors may converge. Additionally, as already discussed, our findings suggest a lack of understanding regarding the varying timeframes required for different resilience factors to develop and change, as well as the degree to which their change can be regulated internally. Regarding the further development of resilience interventions and assuming that resilience factors require more time or external support to develop, so that change becomes better understood, such components could be positioned strategically at the beginning or end of an intervention. For research on mechanisms of change, this underscores the need for a more fine-grained level of investigation of mediators that also respects strict temporal precedence. One promising approach would be to assess the mediating role of resilience factors during the course of the intervention itself. To this end, methods such as session-by-session assessments or ecological momentary assessments in daily life between sessions may be particularly useful [96,97]. An alternative approach could involve using dynamic network models to capture the complexity inherent in change processes within interventions [98].

Third, by targeting multiple resilience factors, our intervention implicitly emphasized the importance of having access to a broad repertoire of resources to draw on various strategies, so individuals are able to respond flexibly to different challenging situations. This idea is reflected in the concept of regulatory flexibility [14], which emphasizes the importance of a good strategy-situation fit, as no single strategy is effective for all stressors. Explicitly integrating elements reflecting such a fit-based perspective into training could be a promising direction for future intervention development, as it could help individuals to learn how to select and apply the most suitable strategies depending on the specific demands of each situation. Examining changes in individuals’ repertoire could be a further meaningful outcome for future intervention studies.

Lastly, given that individualized resilience interventions have not yet been explored sufficiently [11], tailoring the content of RESIST based on individual resource profiles could be a promising avenue for future research. For one study, Fassnacht et al [70] adopted a similar approach by integrating an assessment tool into their intervention, which provided participants with a profile of personal strengths and vulnerabilities to support informed decisions about which areas they should focus on. Building on this idea, incorporating a resilience profile assessment to identify participants’ existing psychosocial resources—upon which the training might then be constructed—could personalize the approach and potentially enhance its effectiveness.

### Practical Implications

The present findings yield several practical implications for implementing digital resilience training, such as RESIST, in occupational settings.

First, adherence to the self-help version of RESIST was low, reflecting a common challenge in digital interventions [99-101]. To enhance adherence, employers should consider recognizing participation in trainings such as RESIST as working time [102]. Low-cost technical measures, such as automated reminders, may also mitigate this issue [103]. More resource-intensive strategies, including personal recruitment, financial incentives, or professional guidance, can further improve engagement and adherence [104,105]. The use of adaptive systems—for example, offering on-demand support during self-help programs or enabling participants in guided formats to opt out of support when preferring independent training—can help limit associated costs. Cost-effectiveness analyses may assist in determining the optimal balance between resource investment to enhance adherence and the resulting health benefits in the population [106].

Second, the observed deterioration rates highlight the need for safety measures, such as providing personal support alongside the intervention or offering clear guidance on where to seek professional external help. Even if the reasons for deterioration remain unclear, as in this study, occupational health care professionals should be aware of potential deterioration and take ethical responsibility into account when offering mental health interventions.

Third, when inviting employees to participate in interventions, occupational health care professionals should communicate potential health benefits appropriately, given the importance of expectations for usage intentions, actual usage, and outcomes [107,108]. The observed response and deterioration rates, together with NNTB and NNTH, provide a solid basis for managing such expectations. Indicators based on standardized mean differences (eg, Cohen *d*) are frequently used but can be difficult for nonresearchers to understand [59] and may not be effective for expectation management.

Fourth, promoting the resilience of individual employees is an important but selective aspect of mental well-being. Interventions such as RESIST should be embedded in a comprehensive occupational health promotion strategy that includes a balanced set of measures addressing both individual and structural, workplace-related aspects [102]. Work-directed interventions—such as improving leadership culture, optimizing workloads, or enhancing effective communication within teams—should complement individual programs. This dual focus helps prevent the impression that employees are solely responsible for their well-being and reinforces the shared responsibility between staff and employers [102].

### Conclusions

This study is among the first to evaluate the effectiveness of a self-help digital resilience intervention that was designed to enhance mental health during stressful times by promoting a set of theory- and evidence-informed selection of resilience factors representing trainable competencies. According to the PASTOR framework, they may serve as correlates of a positive appraisal style of stressors as a higher-order resilience mechanism. The interventional techniques used in the intervention, according to Strengths-Based CBT, proved to be feasible and effective, which confirms the results of the pilot study [17]. Especially when intervention developers find the idea convincing that the methods used to promote resilience should be different from the methods used in psychotherapy aiming at reducing distress, then Strengths-Based CBT appears to be a promising option.

By studying resilience factors as mediators, this trial further addressed the recent call to investigate mechanisms of change within digital resilience interventions [11] to optimize interventional design and outcomes. Mediation analysis suggested that optimism and self-compassion may be important drivers promoting resilient outcomes, while redesigning the intervention seems to be needed to achieve stronger effects for self-efficacy and perceived social support. Future research is needed to investigate the relationships between resilience factors and higher-order resilience mechanisms, the ease of implementation, and the temporal dynamics of how resilience factors exert their protective effects. Finally, a standardized classification system for resilience interventions and a consensus on what constitutes such interventions are urgently needed to enable meaningful comparisons between resilience interventions. This should form the basis of a structured process to improve resilience interventions and make them more effective.

## Supplementary material

10.2196/78335 Multimedia Appendix 1 Information on intervention description according to the TIDieR (Template for Intervention Description and Replication) checklist.

10.2196/78335 Multimedia Appendix 2 Intervention content of the web- and app-based resilience training RESIST.

10.2196/78335 Multimedia Appendix 3 Screenshots of the web- and app-based components of RESIST.

10.2196/78335 Multimedia Appendix 4 Response formats and reliability of patient-reported outcomes measures.

10.2196/78335 Multimedia Appendix 5 Response and deterioration rates for the primary outcome measure of stress.

10.2196/78335 Multimedia Appendix 6 Between-group differences postintervention and at 3-month follow-up for mental health- and work-related secondary outcome measures.

10.2196/78335 Multimedia Appendix 7 Within-subject comparisons for the intervention group from baseline to 6-month follow-up.

10.2196/78335 Multimedia Appendix 8 Additional mediation analyses’ results.

10.2196/78335 Multimedia Appendix 9 Engagement with the web and app components of the intervention.

10.2196/78335 Checklist 1 CONSORT-eHEALTH checklist (V1.6).

## Abbreviations

ANCOVA: analysis of covariance
CBT: cognitive behavioral therapy
CONSORT-eHEALTH: Consolidated Standards of Reporting Trials of Electronic and Mobile Health Applications and Online Telehealth
IG: intervention group
NNTB: numbers needed to treat for benefit
NNTH: numbers needed to treat for harm
PASTOR: Positive Appraisal Style Theory of Resilience
PMRe: personal model of resilience
PSS-10: Perceived Stress Scale-10
RCT: randomized controlled trial
TIDieR: Template for Intervention Description and Replication
WL: waitlist control group
