# Supplementary material for: Effectiveness of and Mechanisms of Change in a Self-Help Web- and App-Based Resilience Intervention on Perceived Stress in the General Working Population: Randomized Controlled Trial
Source: J Med Internet Res. 2026 Jan 5;28:e78335. doi: 10.2196/78335 (PMC12775761; doi:10.2196/78335)
Supplement: Multimedia Appendix 2 — Intervention content of the web- and app-based resilience training RESIST. [file jmir-v28-e78335-s002.docx]

| Intervention  components | Strengths-based CBT content | | | | Further session content of web component |
| --- | --- | --- | --- | --- | --- |
|  | Step | Exercise | Content |  | |
| App | | | | | |
|  | Step 1:  Search for strengths  Step 3+4: Apply/ practice PMRe | *Moments of resilience*  *Power-Ups* | - Collecting past experiences of resilience - Uploading a photo linked to a specific moment - Reflecting on what contributed to resilience in the respective moment - Fostering resilience factor with small exercises | - | |
| Web component | | | | | |
| Session 1 |  |  |  | - Introduction into the intervention - Reflecting on personal intervention motivation | |
| Session 2 | Step 2:  Construct PMRe  Step 3+4:  Apply/practice PMRe | *Resilience self-image*  *Resilience project* | - Developing a positive self-image including metaphors on basis of selected *moment of resilience* - Selecting future challenge in need of resilience - Planning how to use *resilience self-image* and resilience factors to overcome challenge - Fostering resilience factor with small exercises | Exercises on fostering self-efficacy:   - Reflecting past achievements - Practicing unconditional appreciation of oneself - Planning daily exercises enhancing self-esteem | |
| Session 3 |  |  |  | Exercises on fostering optimism:   - Practicing an optimistic perspective - Practicing a positive attributional style | |
| Session 4 |  |  |  | Exercises on fostering social support:   - Reflecting current social relationships - Setting goals to improve relationships | |
| Session 5 |  |  |  | Exercises on fostering self-compassion:   - Identifying self-critical thoughts - Replacing self-critical thoughts with self-compassionate thoughts - Reflecting life-domain-balance | |
| Session 6 |  |  |  | - Review of sessions’ content and personal summaries - Writing a personal letter to future self | |
