# Supplementary material for: Effectiveness of and Mechanisms of Change in a Self-Help Web- and App-Based Resilience Intervention on Perceived Stress in the General Working Population: Randomized Controlled Trial
Source: J Med Internet Res. 2026 Jan 5;28:e78335. doi: 10.2196/78335 (PMC12775761; doi:10.2196/78335)
Supplement: Multimedia Appendix 3 — Screenshots of the web- and app-based components of RESIST. [file jmir-v28-e78335-s003.docx]

**Figure S1.** Web-component.


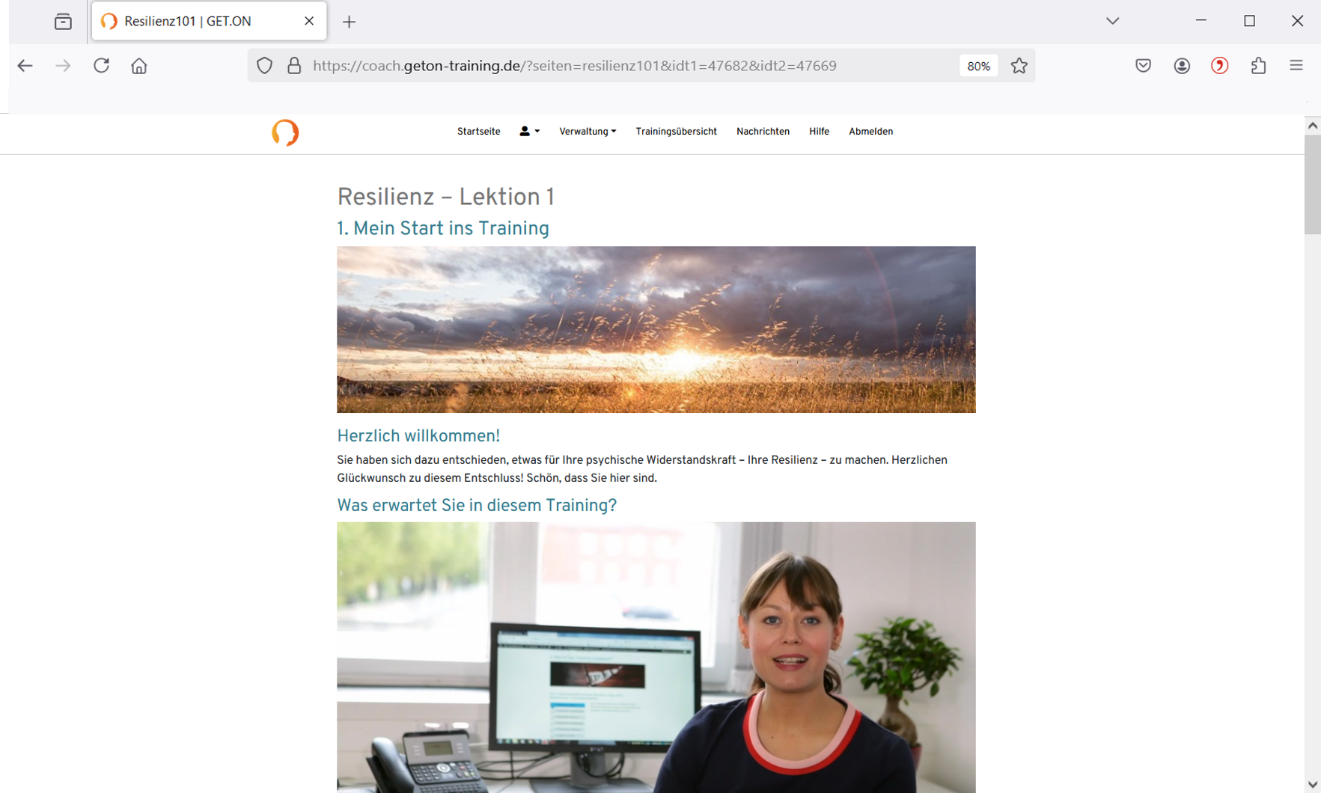


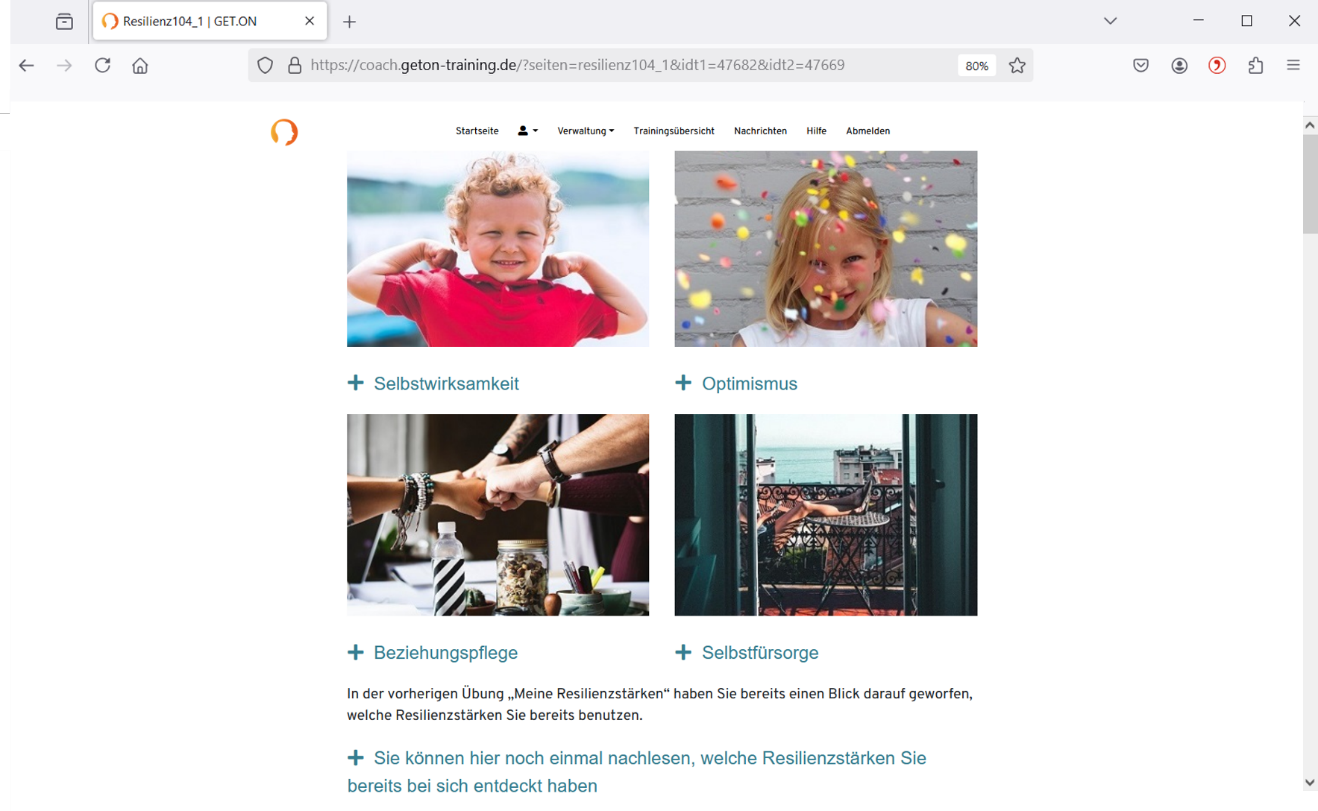


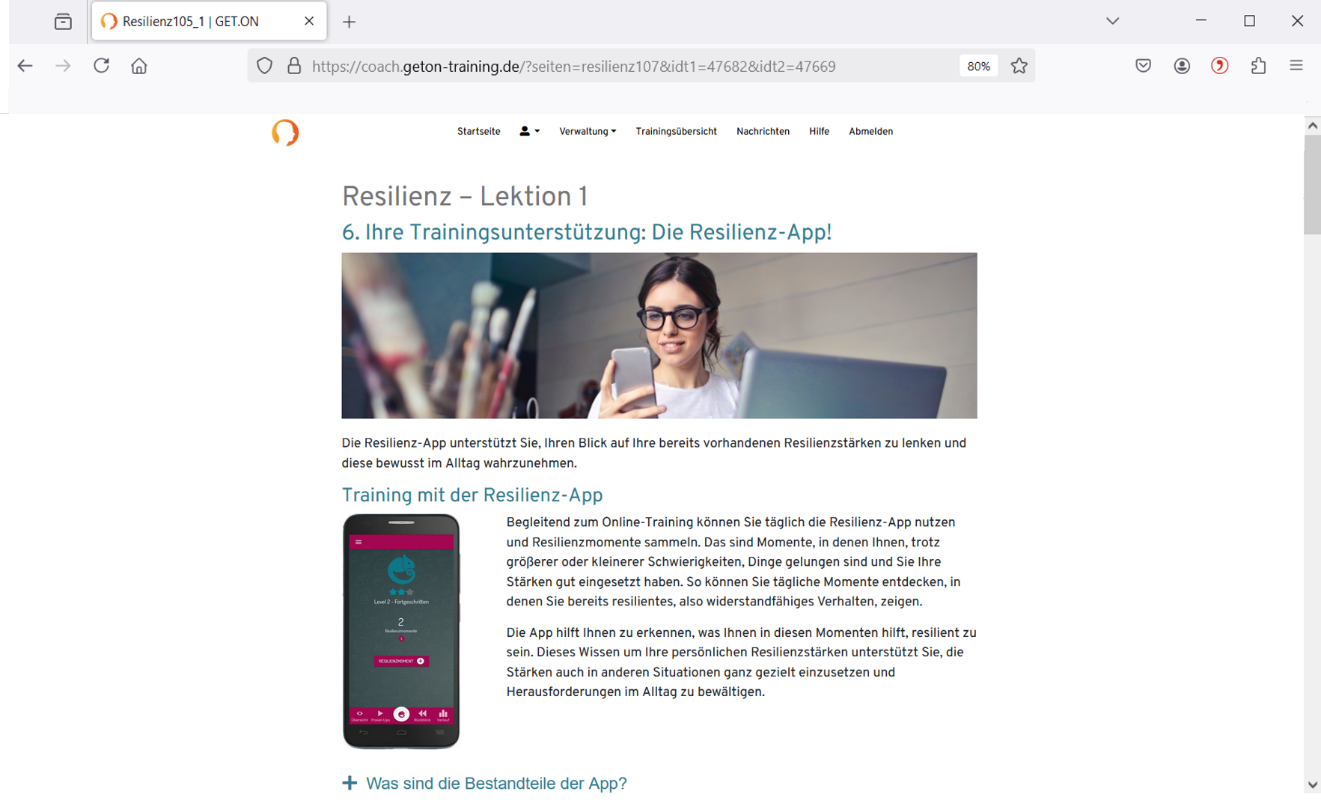

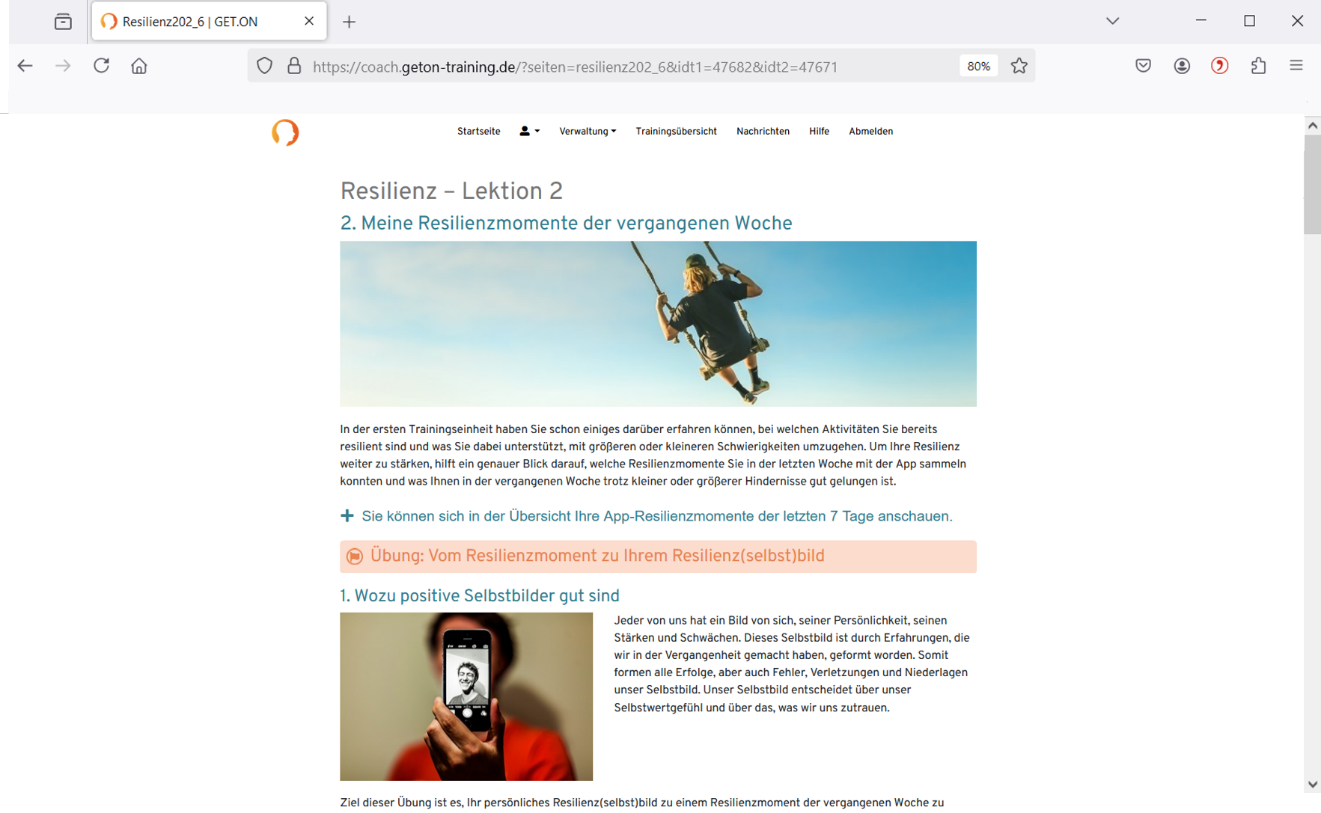


**Figure S2.** App component.

a.
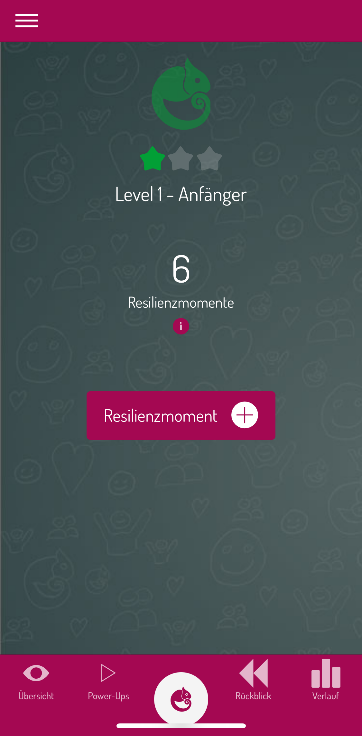
 b.
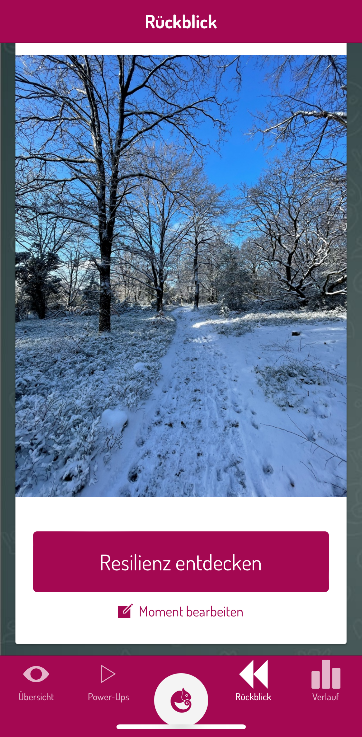
 c.
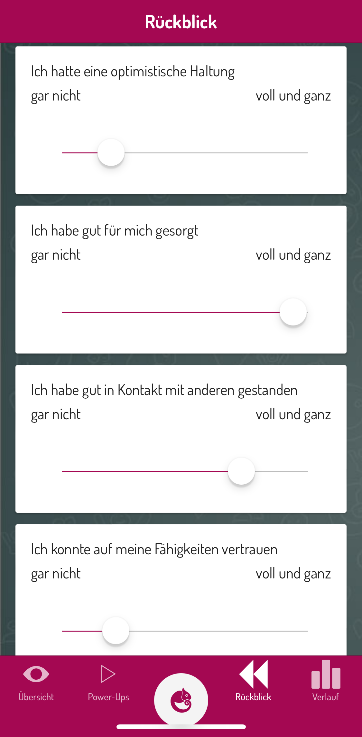


d.
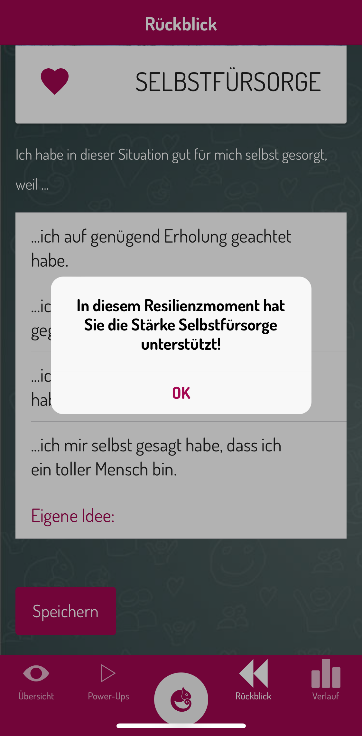
 e.
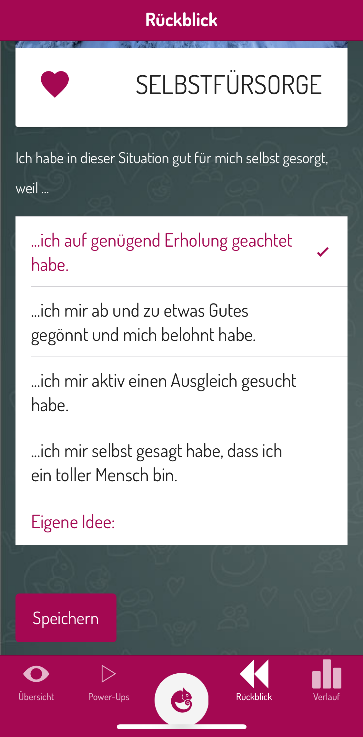
 f.
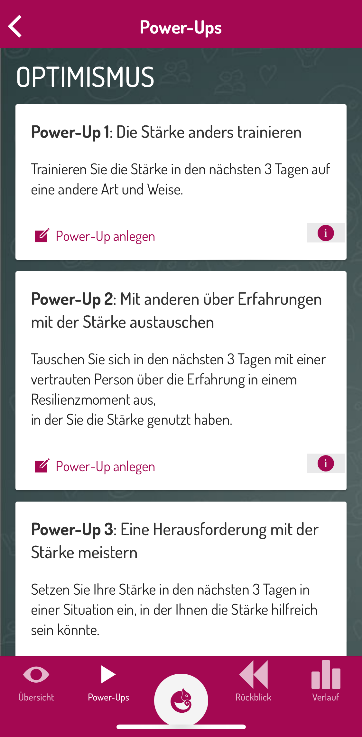


a. Screenshot of the app’s home screen with button to collect *moment of resilience*; b. screenshot of uploaded photo linked to *moment of resilience*; c. screenshot of review and rating of resilience factors present in the respective *moment of resilience*, d. screenshot of assignment of moment of resilience to one of the four resilience factors; e. review of behaviors and cognitions that contributed to resilience in *moment of resilience*, f. screenshot of planning a *power-up.*
