# Supplementary material for: Effectiveness of and Mechanisms of Change in a Self-Help Web- and App-Based Resilience Intervention on Perceived Stress in the General Working Population: Randomized Controlled Trial
Source: J Med Internet Res. 2026 Jan 5;28:e78335. doi: 10.2196/78335 (PMC12775761; doi:10.2196/78335)
Supplement: Multimedia Appendix 4 — Response formats and reliability of patient-reported outcomes measures. [file jmir-v28-e78335-s004.docx]

| Measure | No. of items | Response format | Reliability (Cronbach α) at baseline |
| --- | --- | --- | --- |
|  |  |  |  |
| Perceived Stress Scale | 10 | 0-4 | .89 |
| Brief Resilience Scale | 6 | 1-5 | .85 |
| General Self-Efficacy Short Scale | 3 | 1-5 | .85 |
| Short Scale for the Assessment of Locus of Control – subscale Internal Control Beliefs | 2 | 1-5 | .68 |
| Short Scale for the Assessment of Locus of Control – subscale External Control Beliefs | 2 | 1-5 | .58 |
| Life Orientation Test | 10 | 0-4 | .81 |
| Self-Compassion Scale Short Form | 12 | 1-5 | .89 |
| Berlin Social Support Scales – subscale Perceived Social Support | 8 | 1-4 | .92 |
| Berlin Social Support Scales – subscale Support Seeking | 5 | 1-4 | .87 |
|  |  |  |  |
| Epidemiologic Studies Depression Scale | 15 | 0-3 | .89 |
| Brief Symptom Inventory | 18 | 0-4 | .87 |
| Life History Calendar | 27 | Life-time  occurrence | - |
| Mainz Inventory of Microstressors | 58 | Occurrence per item within week (1-7) | - |
| Effort-Reward-Imbalance Questionnaire – subscale Efforts | 3 | 1-4 | .80 |
| Effort-Reward-Imbalance Questionnaire – subscale Rewards | 7 | 1-4 | .75 |
| Effort-Reward-Imbalance Questionnaire – subscale Overcommitment | 6 | 1-4 | .79 |
| Trimbos and Institute of Medical Technology Assessment Cost Questionnaire for Psychiatry –Absenteeism item | 1 | Days of absenteeism within past 28 days | - |
| Trimbos and Institute of Medical Technology Assessment Cost Questionnaire for Psychiatry –Presenteeism item | 1 | Days of presenteeism within past 28 days | - |
| Work Ability Index | 1 | 0-10 | - |
| Client Satisfaction Questionnaire | 8 | 0-3 | .95 |
| AttrakDiff | 10 | 1-7 | .89 |
