# Supplementary material for: Effectiveness of and Mechanisms of Change in a Self-Help Web- and App-Based Resilience Intervention on Perceived Stress in the General Working Population: Randomized Controlled Trial
Source: J Med Internet Res. 2026 Jan 5;28:e78335. doi: 10.2196/78335 (PMC12775761; doi:10.2196/78335)
Supplement: Multimedia Appendix 5 — Response and deterioration rates for the primary outcome measure of stress. [file jmir-v28-e78335-s005.docx]

| Criterion | T2^a^ | | | | | T3^b^ | | | |
| --- | --- | --- | --- | --- | --- | --- | --- | --- | --- |
|  | NNTB^c/^  NNTH^d^ | 95% CI | IG^e^ | WL^f^ | NNTB/  NNTH | | 95% CI | IG | WL |
|  |  |  |  |  |  | |  |  |  |
| 20 % improvement | 3.87 | 2.80 to 6.31 | 96 (55%) | 50 (29%) | 3.42 | | 2.56 to 5.16 | 99 (56%) | 47 (27%) |
| Reliable improvement | 4.35 | 3.05 to 7.58 | 85 (48%) | 44 (25%) | 3.62 | | 2.70 to 5.50 | 83 (47%) | 34 (19%) |
| Remission^g^ | 5.00 | 3.13 to 12.39 | 64 (50%) | 36  (30%) | 6.58 | | 3.68 to 31.17 | 60 (47%) | 38 (32%) |
| 20 % deterioration | 8.20 | 5.01 to 22.52 | 19 (11%) | 40 (23%) | 7.51 | | 4.80 to 17.21 | 16 (9%) | 39 (22%) |
| Reliable deterioration | 10.78 | 6.30 to 37.52 | 12 (7%) | 28 (16%) | 10.81 | | 6.57 to 30.57 | 8 (5%) | 24 (14%) |

^a^T2: Post-intervention (8 weeks after randomization)

^b^T3: 3-months follow-up (3 months after randomization)

^c^NNTB: Numbers needed to treat (beneficial outcome)

^d^NNTH: Numbers needed to treat (harmful outcome)

^e^IG: Intervention group

^f^WL: Waitlist control group
^g^N:248 (reduced sample without participants scoring ≤ 17 at baseline)
