# Supplementary material for: Effectiveness of and Mechanisms of Change in a Self-Help Web- and App-Based Resilience Intervention on Perceived Stress in the General Working Population: Randomized Controlled Trial
Source: J Med Internet Res. 2026 Jan 5;28:e78335. doi: 10.2196/78335 (PMC12775761; doi:10.2196/78335)
Supplement: Multimedia Appendix 6 — Between-group differences postintervention and at 3-month follow-up for mental health- and work-related secondary outcome measures. [file jmir-v28-e78335-s006.docx]

| Outcome | Differences between intervention group and waitlist control group | | | | | |
| --- | --- | --- | --- | --- | --- | --- |
|  | T2^a^ | | | T3^b^ | | |
|  | *F* test (*df*) | *P* value | Cohen *d* (95% CI) | *F* test (*df*) | *P* value | Cohen *d* (95% CI) |
| **Secondary outcomes** | | | | | | |
| ***Mental health*** | | | | | | |
| Depressive symptoms | 7.80 (1, 189) | .006 | -0.24 (-0.44 to -0.05) | 2.25 (_1, 66)_ | .14 | -0.16 (-0.41 to 0.10) |
| Psychological distress | - ^c^ | - ^c^ | - ^c^ | 1.28 (1, 114) | .26 | -0.13 (-0.39 to 0.12) |
| ***Work-related health*** | | | | | | |
| Work ability | 4.83 (1, 119) | .03 | 0.28 (0.05 to 0.51) | 0.79 (1, 57) | .38 | 0.04 (-0.41 to 0.49) |
| Effort | - ^c^ | - ^c^ | - ^c^ | 10.81 (1, 202) | .001 | -0.26 (-0.45 to -0.06) |
| Reward | - ^c^ | - ^c^ | - ^c^ | 2.80 (1, 60) | .10 | 0.06 (-0.21 to 0.34) |
| Over-commitment | 5.48 (1, 87) | .02 | -0.22 (-0.42 to -0.02) | 17.48 (1, 135) | <.001 | -0.36 (- 0.53 to -0.18) |
| Absenteeism | 1.66 (1, 37) | .21 | -0.20 (-0.60 to 0.19) | 1.38 (1, 40) | .25 | -0.18 (-0.71 to 0.35) |
| Presenteeism | 0.70 (1, 45) | .41 | -0.02 (-0.51 to 0.48) | 1.72 (1, 34) | .20 | 0.26 (-0.38 to 0.90) |

^a^T2: Post-intervention (8 weeks after randomization)

^b^T3: 3-months follow-up (3 months after randomization)

^c^Missing data due to data management errors
