# Supplementary material for: Effectiveness of and Mechanisms of Change in a Self-Help Web- and App-Based Resilience Intervention on Perceived Stress in the General Working Population: Randomized Controlled Trial
Source: J Med Internet Res. 2026 Jan 5;28:e78335. doi: 10.2196/78335 (PMC12775761; doi:10.2196/78335)
Supplement: Multimedia Appendix 7 — Within-subject comparisons for the intervention group from baseline to 6-month follow-up. [file jmir-v28-e78335-s007.docx]

| Outcome | Differences within intervention group | | |
| --- | --- | --- | --- |
|  | T4^a^ | | |
|  | *F* test (*df*) | *P* value | Cohen *d* (95% CI) |
| **Primary outcome** |  |  |  |
| Stress | 128.6 (1, 175) | <.001 | -1.01 (-1.25 to -0.77) |
| **Secondary outcomes** |  |  |  |
| Resilience | 187.4 (1, 175) | <.001 | 1.19 (0.90 to 1.48) |
| ***Resilience factors*** |  |  |  |
| Self-efficacy | 86.12 (1, 175) | <.001 | 0.80 (0.55 to 1.04) |
| Internal control | 13.56 (1, 175) | <.001 | 0.32 (0.03 to 0.61) |
| External control | 52.54 (1, 175) | <.001 | -0.67 (-0.95 to -0.39) |
| Optimism | 111.7 (1, 175) | <.001 | 0.87 (0.58 to 1.16) |
| Self-compassion | 240.3 (1, 175) | <.001 | 1.25 (0.95 to 1.54) |
| Perceived support | 17.34 (1, 175) | <.001 | 0.37 (0.09 to 0.65) |
| Support seeking | 47.77 (1, 175) | <.001 | 0.60 (0.27 to 0.93) |
| ***Mental health*** |  |  |  |
| Depressive symptoms | 7.51 (1, 175) | .007 | -0.25 (-0.52 to 0.02) |
| Psychological distress | -^b^ | -^b^ | -^b^ |
| ***Work-related health*** |  |  |  |
| Work ability | 4.84 (1, 175) | .029 | 0.20 (-0.07 to 0.47) |
| Effort | -^b^ | -^b^ | -^b^ |
| Reward | -^b^ | -^b^ | -^b^ |
| Over-commitment | 100.2 (1, 175) | <.001 | -0.81 (-1.14 to -0.49) |
| Absenteeism | 0.87 (1, 175) | .35 | 0.04 (-0.91 to 0.98) |
| Presenteeism | 46.42 (1, 175) | <.001 | -0.55 (-1.28 to 0.19) |

^a^T4: 6-months follow-up (6 months after randomization)
^b^Missing due to data management errors
