# Supplementary material for: Effectiveness of and Mechanisms of Change in a Self-Help Web- and App-Based Resilience Intervention on Perceived Stress in the General Working Population: Randomized Controlled Trial
Source: J Med Internet Res. 2026 Jan 5;28:e78335. doi: 10.2196/78335 (PMC12775761; doi:10.2196/78335)

**Figure S1.** Parallel multiple mediation model with 3-month follow-up (T3) self-perceived resilience scores as the outcome variable (Y), post-treatment (T2) resilience factors scores as mediators and baseline values of mediators and outcome as covariates. Intervention (X) is coded 1= intervention group (IG), 0= waitlist control group (WL). Unstandardized beta coefficients are shown with 95% CIs in brackets.

**
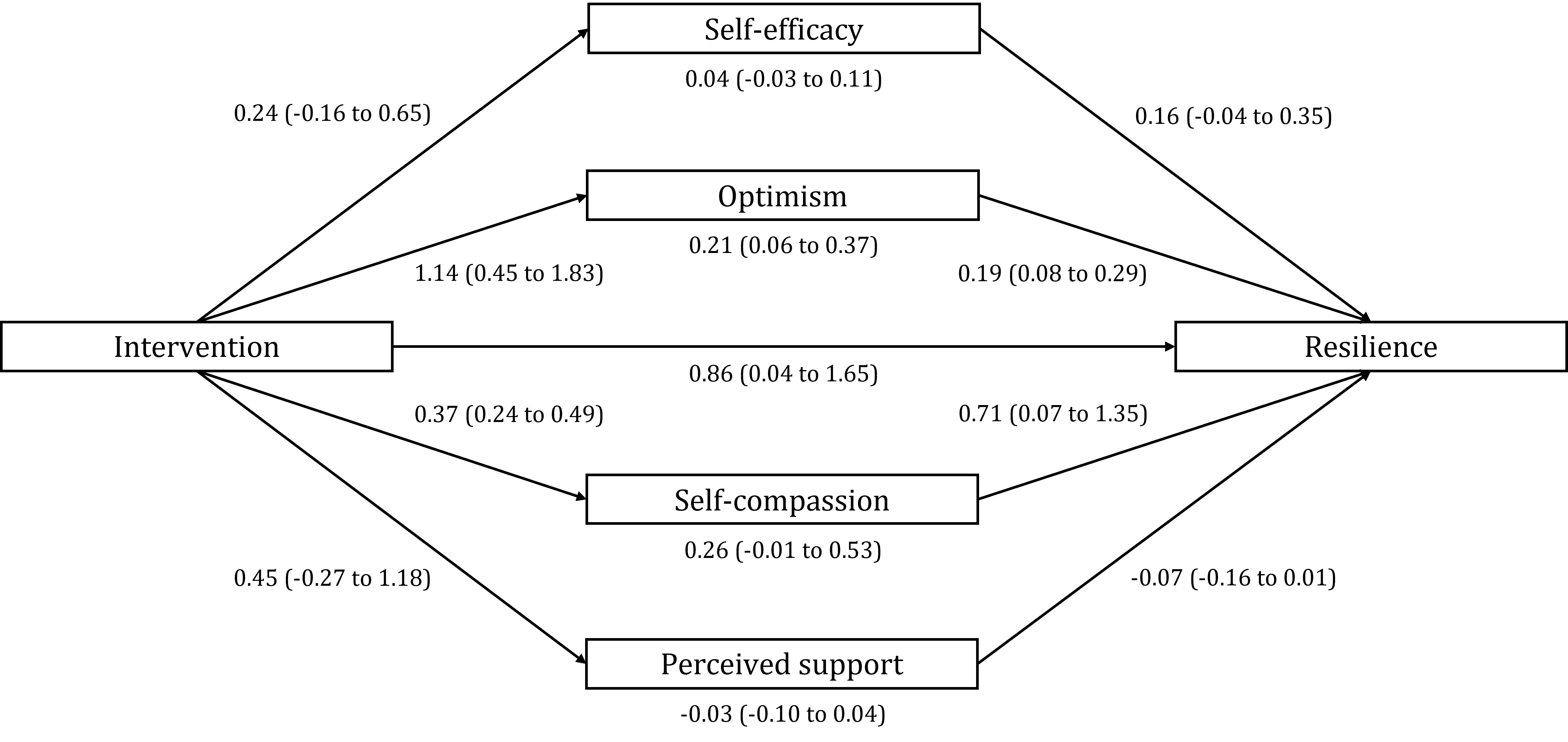
**

**Figure S2.** Parallel multiple mediation model conducted as sensitivity analysis within study completer sample with 3-month follow-up (T3) stress scores as the outcome variable (Y), post-treatment (T2) resilience factors scores as mediators and baseline values of mediators and outcome as covariates. Intervention (X) is coded 1= intervention group (IG), 0= waitlist control group (WL). Unstandardized beta coefficients are shown with 95% CIs in brackets.


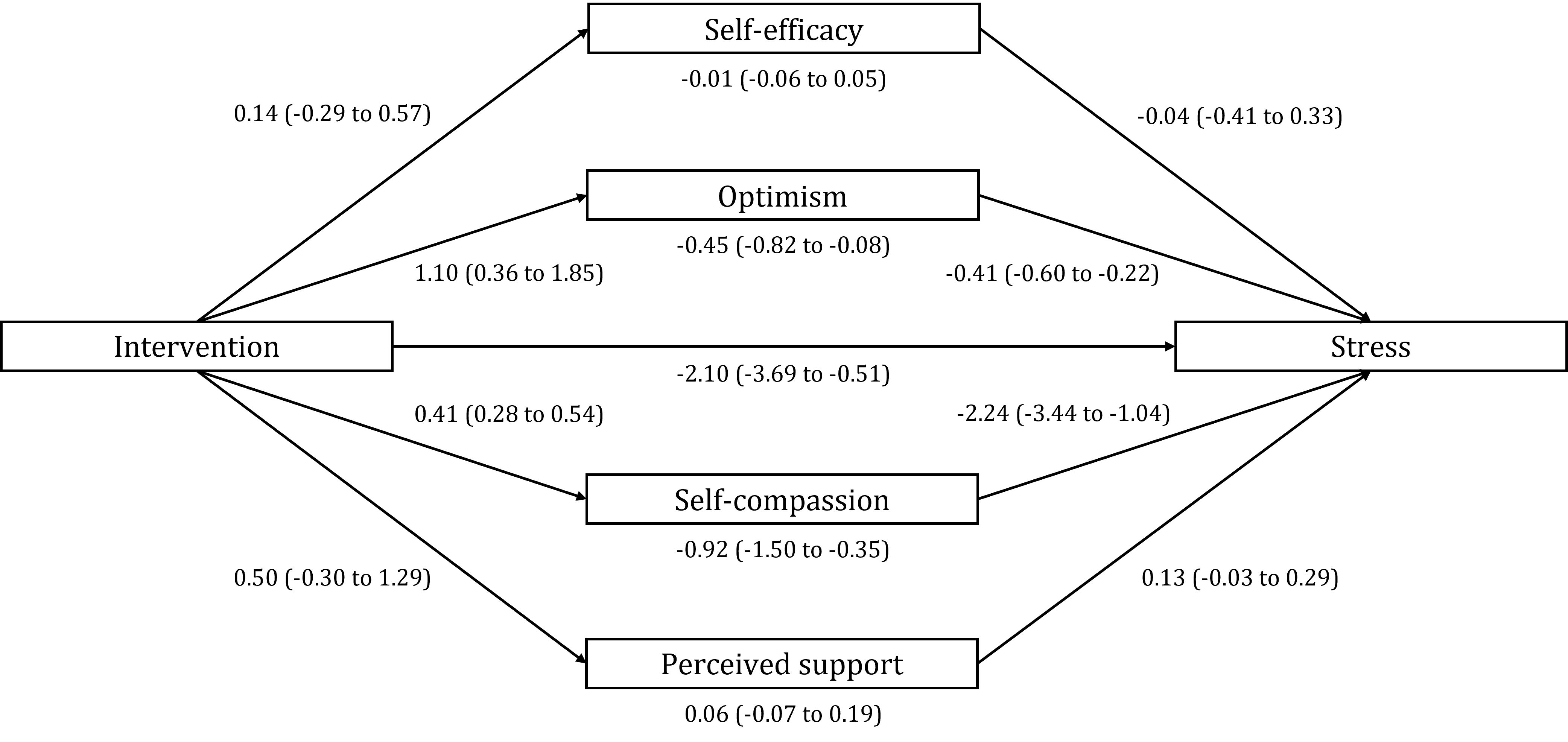


**Figure S3.** Parallel multiple mediation model conducted as sensitivity analysis within study completer sample with 3-month follow-up (T3) self-perceived resilience scores as the outcome variable (Y), post-treatment (T2) resilience factors scores as mediators and baseline values of mediators and outcome as covariates. Intervention (X) is coded 1= intervention group (IG), 0= waitlist control group (WL). Unstandardized beta coefficients are shown with 95% CIs in brackets.


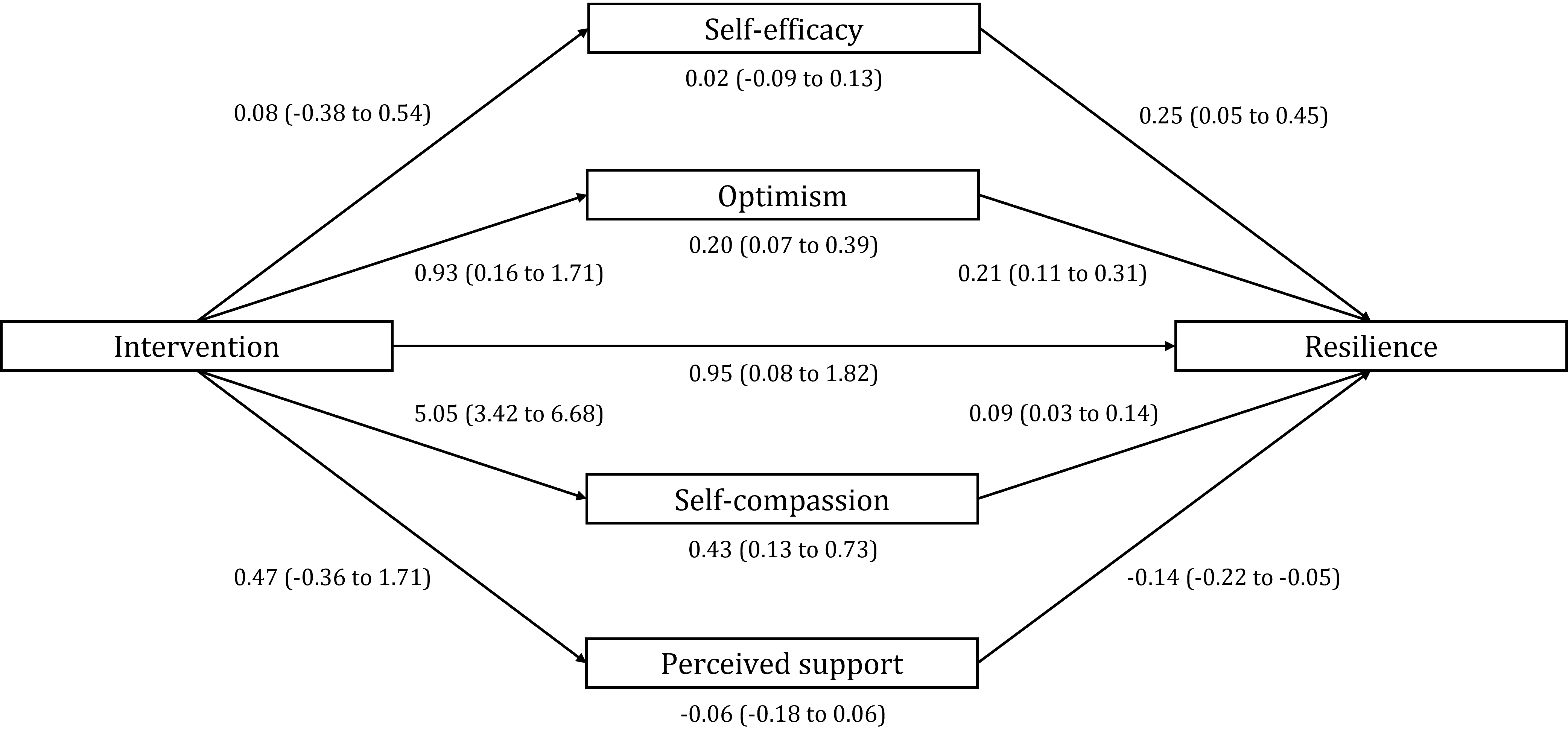

Supplement: Multimedia Appendix 8 — Additional mediation analyses’ results. [file jmir-v28-e78335-s008.docx]
