# Supplementary material for: Effectiveness of and Mechanisms of Change in a Self-Help Web- and App-Based Resilience Intervention on Perceived Stress in the General Working Population: Randomized Controlled Trial
Source: J Med Internet Res. 2026 Jan 5;28:e78335. doi: 10.2196/78335 (PMC12775761; doi:10.2196/78335)
Supplement: Multimedia Appendix 9 — Engagement with the web and app components of the intervention. [file jmir-v28-e78335-s009.docx]

| Intervention component | Participants, n (%) | Completion rates and engagement details |
| --- | --- | --- |
|  |  |  |
| Web | 156 (88.6%) started web component | Session 1: 110 (70.5%) |
|  |  | Session 2: 74 (47.4%) |
|  |  | Session 3: 53 (40.0%) |
|  |  | Session 4: 45 (28.8%) |
|  |  | Session 5: 39 (25.0%) |
|  |  | Session 6: 29 (18.6%) |
|  | 20 (11.4%) did not start web component |  |
| App | 104 (59.1%) used app | Self-compassion: 1122 moments |
|  |  | Social support: 811 moments |
|  |  | Self-efficacy: 624 moments |
|  |  | Optimism: 507 moments |
|  | 72 (40.9%) did not use app component |  |
